# MicroRNA-19b-3p dysfunction of mesenchymal stem cell-derived exosomes from patients with abdominal aortic aneurysm impairs therapeutic efficacy

**DOI:** 10.1186/s12951-023-01894-3

**Published:** 2023-04-26

**Authors:** Yuxiao Zhang, Xiaoran Huang, Tucheng Sun, Linli Shi, Baojuan Liu, Yimei Hong, Qing-Ling Fu, Yuelin Zhang, Xin Li

**Affiliations:** 1 School of Medicine, South China University of Technology, Guangzhou, China; 2 Department of Emergency Medicine, Guangdong Provincial People’s Hospital (Guangdong Academy of Medical Sciences), Southern Medical University, Guangzhou, Guangdong China; 3 Otorhinolaryngology Hospital, The First Affiliated Hospital, Sun Yat-sen University, Guangzhou, China

**Keywords:** Mesenchymal stem cells, Exosomes, Abdominal aortic aneurysm, Vascular smooth muscle cells, Senescence

## Abstract

Senescence of vascular smooth muscle cells (VSMCs) contributes to the formation of abdominal aortic aneurysm (AAA). Although mesenchymal stem cell exosomes (MSC-EXO) have been confirmed to restrict the development of AAA, their biological activity depends largely on the physiological state of the MSCs. This study aimed to compare the effects of adipose-derived MSC-EXO from healthy donors (HMEXO) and AAA patients (AMEXO) on senescence of VSMCs in AAA and explore the underlying mechanisms. An *ApoE-/-* mouse model of AAA was used to investigate the therapeutic effects of HMEXO, AMEXO or miR-19b-3p-AMEXO on AAA development. This in vitro model of AAA was established by treating VSMCs with Ang II (Angiotensin II). The senescence of VSMCs was determined by senescence-associated β-galactosidase (SA-β-gal) staining. The morphology of mitochondria in VSMCs was examined by MitoTracker staining. HMEXO exhibited superior capacity compared with AMEXO to inhibit VSMC senescence and attenuate AAA formation in Ang II–treated *ApoE-/-* mice. In vitro, both AMEXO and HMEXO inhibited Ang II-induced VSMC senescence via downregulation of mitochondrial fission. Notably, compared with HMEXO, the ability of AMEXO to inhibit VSMC senescence was significantly decreased. miRNA sequencing and the expression of miR-19b-3p was significantly decreased in AMEXO compared with HMEXO. Luciferase assay suggested that MST4 (Mammalian sterile-20-like kinase 4) is a potential target of miR-19b-3p. Mechanistically, miR-19b-3p in HMEXO ameliorated VSMC senescence by inhibiting mitochondrial fission via regulation of the MST4/ERK/Drp1 signaling pathway. Overexpression of miR-19b-3p in AMEXO improved their beneficial effect on AAA formation. Our study reveals that MSC-exosomal miR-19b-3p exerts protective effects against Ang II-induced AAA and VSMC senescence via regulation of the MST4/ERK/Drp1 pathway. The pathological state of AAA patients alters the miRNA components of AMEXO and impairs their therapeutic benefits.

**Supplementary Information:**

The online version contains supplementary material available at 10.1186/s12951-023-01894-3.

## Introduction

Abdominal aortic aneurysm (AAA) is an age-related disease characterized by an aortic diameter that is dilated to more than 1.5 times its normal value [1]. Rupture of AAA is a major cause of morbidity and mortality worldwide [2]. Despite advanced surgical and medical interventions, none has been shown to reduce the risk of rupture [3, 4]. A novel treatment is urgently needed.

Cellular senescence is a permanent barrier to proliferation in response to various stresses and closely associated with the development of age-related diseases [5, 6]. It has been well documented that vascular smooth muscle cell (VSMC) senescence contributes to AAA formation [7–9]. Mitochondrial dysfunction caused by a mitochondrial fusion and fission imbalance plays a critical role in regulating cellular senescence [10]. Dynamin-related protein 1 (Drp1) is a member of the GTPase family and is mainly responsible for driving fission of mitochondria. Previous studies have shown that excessive aortic mitochondrial fission manifested by increased phosphorylation of Drp1 leads to VSMC injury, ultimately resulting in AAA formation [11]. Moreover, it has been reported that Ang II induces premature VSMC senescence by stimulating mitochondrial fission and superoxide generation [12]. Our previous study demonstrated that VSMCs isolated from AAA patients were senescent, displaying elevated reactive oxide species (ROS) and mitochondrial dysfunction [13]. Nonetheless the mechanisms underlying Ang II-induced VSMC senescence in AAA formation via regulation of mitochondrial fission remain unclear.

Over the past two decades, mesenchymal stem cell (MSC)-based therapy has shown promising results in various cardiovascular diseases. Intravenous administration of adipose tissue-derived MSCs (ADMSCs) has been shown to inhibit Ang II-induced AAA formation in *ApoE-/-* mice [14]. Nonetheless stem cell therapy carries a risk of promoting tumor growth and teratoma formation [15, 16]. The therapeutic effects of MSCs are attributed mainly to their paracrine effects. There is evidence that MSC-derived exosomes (MSC-EXO) have the potential to effectively inhibit cellular senescence in cardiovascular disease [17–20]. Nonetheless exosomes (EXO)-mediated therapeutic effects are largely dependent on their components, especially miRNAs that are largely regulated by the physiological state of their parent cell [21]. It has been reported that the reduced miR-21-5p in cardiac stromal cell-derived EXO from patients with heart failure impairs their regenerative potential [22]. Remarkably, our previous study showed that MSCs isolated from patients with AAA exhibited senescent phenomena leading to decreased function [23]. Whether the anti-senescent ability of MSC-EXO isolated from AAA patients (AMEXO) is impaired has not been validated, and the mechanism remains to be elucidated. We compared the therapeutic effects of adipose-derived MSC-EXO from healthy donors (HMEXO) and AMEXO on AAA formation and explored the underlying molecular mechanisms. Compared with AMEXO, HMEXO enriched with miR-19b-3p more effectively attenuated AAA formation and inhibited mitochondrial fission and ROS generation in senescent VSMCs by regulating the MST4/ERK/Drp1 pathway. Overexpression of miR-19b-3p in AMEXO significantly improved their protective effects against AAA formation in mice.

## Materials and methods

### Cell culture

Subcutaneous adipose tissue was harvested from AAA patients during aortic repair surgery (n = 3) and age-matched healthy donors (n = 3). Their demographic information is summarized in Supplementary Table 1. All participants provided written informed consent. This study was approved by the research ethics board of Guangdong Provincial People’s Hospital (No. GDREC2018060H). ADMSCs were prepared and cultured as previously reported [23]. Briefly, adipose tissue was cut into small pieces and subjected to enzymatic digestion for 1 h at 37 ℃, followed by filtration of the undigested tissue pieces and centrifugation to obtain cell precipitates that were plated on 10 cm culture dishes. After 48 h, non-adherent cells were removed and the remaining MSCs cultured in Dulbecco’s modified eagle medium (DMEM; 11,965,084, Gibco) infused with 10% fetal bovine serum (10,099,141, Gibco), NEAA (11,140,050, Gibco) and 0.1 mM 2-mercaptoethanol (21,985,023, Gibco). VSMCs (n = 3 independent samples) were isolated from the abdominal aortic tissue of 3 healthy donors and 3 adult male mice as previously reported [13]. 293T cells used in this study were prepared as described in our previous study, [24] and cultured in DMEM medium supplemented with 10% fetal bovine serum. All cells were passaged every 3–4 days at approximately 80–90% confluence. ADMSCs and VSMCs at passage 3–4 were used in the current study.

### Isolation and characterization of MSC-EXO

HMEXO and AMEXO were isolated from ADMSCs derived from healthy and AAA patients, respectively. Briefly, EXO-rich medium was prepared using chemically defined protein-free (CDPF) medium containing chemically defined medium for Chinese hamster ovary (CD-CHO; 10,743,029, Gibco), HT supplement (11,067,030, Gibco), L-Glutamine (25,030,081, Gibco), D-(+)-glucose (A2494001, Gibco), NEAA and vitamin solution (11,120,052, Gibco), and EXO were isolated by differential ultracentrifugation as previously reported [25, 26]. Briefly, MSCs at 70–80% confluence were washed three times with phosphate-buffered saline(PBS) and the medium changed to CDPF. CDPF medium was discarded after 6 h of incubation and replaced with fresh CDPF medium. Forty-two hours later, CDPF medium was collected and centrifuged at 10 000 g for 30 min to remove cell debris. Next, cell-free supernatant was centrifuged at 100 000 g (Optima XPN-100; Beckman Coulter) at 4 °C for at least 70 min to obtain HMEXO and AMEXO. HMEXO and AMEXO were washed with PBS and centrifugation repeated once under the same conditions, then EXO were resuspended in PBS and stored at -80 ℃. The protein concentration of HMEXO and AMEXO was evaluated by bicinchoninic acid protein assay. Mean particle size and concentration were quantified by nanoparticle tracking analysis (NanoSight NS300; Malvern). EXO were identified using transmission electron microscopy (H-7650; Hitachi H-7650). Surface markers of EXO including CD63, CD81, TSG101 and the negative marker of Calnexin were detected by Western blotting.

### Animal study

All experimental animal procedures in this study were approved by the Ethics Committee of South China University of Technology (No. 2,020,008) and performed in accordance with the National Institutes of Health Guide for the Care and Use of Laboratory Animals. To avoid the protective effect of estrogen, the AAA model was established in male mice as previously described [27]. Briefly, 8 to 10-week-old *ApoE-/-* male mice on a C57BL/6J background were purchased from GemPharmatech Co., Ltd. and maintained in specific pathogen-free conditions and fed either control diet major containing protein (25%), carbohydrate (30%) and fat (4.5%) (10,010, Beijing Boaigang Biological Technology) or a high-fat diet containing protein (25%), carbohydrate (30%) and fat (45%) (D12451, Beijing Boaigang Biological Technology). Considering the death caused by aortic aneurysm rupture and reducing the number of animals used, fifty age-matched male *ApoE-/-* mice were divided into five groups (n = 10/group) by a random number table according to body weight, age, blood pressure. For Ang II infusion, mice were anesthetized with intraperitoneal pentobarbital sodium (60 mg/kg) and an Alzet osmotic pump (Alzet, model 2004; DURECT Corp.) implanted through an incision in the dorsum. Ang II (HY-13,948, MCE) at a release rate of 1.44 mg/kg/day was infused by minipump for 4 weeks. Another group of mice that received a saline infusion served as the sham group. To investigate the therapeutic effect of MSC-EXO on AAA, Ang II-treated mice were intravenously injected with HMEXO (MSC-EXO from 3 healthy donors), AMEXO (MSC-EXO from 3 AAA patients) or miR-19b-3p-AMEXO (50 µl PBS containing 5 × 10^9^ EXO) every three days starting the day following minipump implantation.

### Ultrasound measurement of aneurysm diameter

After 28 days of Ang II perfusion, B-mode ultrasound imaging of the abdominal aorta using a Vevo 2100 high-resolution imaging system (40-MHz transducer; Visualsonics) was carried out as previously described [28]. Briefly, mice were anesthetized with 3% isoflurane and the location of the abdominal aorta identified from start to finish by an experienced vascular sonographer who was blind to the grouping of mice. Cine-loops of 100 frames were acquired by the renal region of the abdominal aorta and used to determine maximal diameters of the abdominal aorta in the suprarenal region independently by two blinded investigators. Ex vitro measurements of the maximum diameter of the abdominal aortas were performed using a vernier caliper to confirm the accuracy of ultrasound measurements.

### Histological and immunohistochemical analysis

The aorta of mice was collected as described previously [11]. After measurement of the aneurysm, all mice were anesthetized with 3% isoflurane and euthanized by exsanguination. The chest and abdominal cavities were opened and the whole aorta removed and photographed with a digital camera. Next, aortic tissue was fixed in 4% paraformaldehyde or snap-frozen in liquid nitrogen and preserved at -80 °C. To assess aortic wall thickness, abdominal aortic tissue was paraffin-embedded and serially sectioned into 5 μm cross-sections. Sections were stained with hematoxylin-eosin (HE) according to the manufacturer’s instructions (C0105S, Beyotime) and photographed using a microscope. Image-Pro Plus software was used to measure different parts of the aortic wall and the average thickness calculated. To investigate the internalization of EXO in mouse aortic wall, abdominal aortic tissue of 6 AAA, 24 h after intravenous injection with 5 × 10^9^ DiI red fluorescent dye (C1036, Beyotime) labeled HMEXO (DiI-HMEXO), DiI labeled AMEXO (DiI-AMEXO) or DiI labeled miR-19b-3p-AMEXO (DiI-miR-19b-3p-AMEXO AMEXO), was also collected and preserved at -80 °C. Subsequently, abdominal aortic tissue from at least 6 mice per group was cut into 5-µm cross-sections and incubated with anti-Ki67 (Abcam, ab15580), anti-MMP-9 (Abcam, ab58803), anti-CD68 (Abcam, ab213363) and anti-α-SMA (BM0002, Boster Biological Technology) at 4 °C overnight. Next, sections were incubated with secondary antibodies for 1 h at room temperature followed by cell nuclei staining using 4′, 6-diamidino-2-phenylindole (DAPI). Images were obtained using a fluorescent microscope. Six random fields per tissue were photographed by fluorescence microscopy (TI-S, Nikon) and mean values used in subsequent statistical analysis.

### VSMCs co-cultured with EXO

In the in vitro study design, VSMCs (n = 3 independent samples) were co-cultured with MSC-EXO from 3 healthy donors (n = 3) or MSC-EXO from 3 AAA patients (n = 3) and comprised the following groups: VSMCs + Ang II + HMEXO cultured for 48 h; VSMCs + DiI-HMEXO cultured for 3 h; VSMCs + Ang II + anti-miR-19b-3p-HMEXO cultured for 48 h; VSMCs + Ang II + AMEXO cultured for 48 h; VSMCs + DiI-AMEXO cultured for 3 h; VSMCs + Ang II + miR-19b-3p-AMEXO. The above VSMCs samples were detected by SA-β-gal assay, western blotting, quantitative real-time polymerase chain reaction (qRT-PCR), MitoTracker staining, Mito-sox staining, and immunocytochemistry. 6 independent replicate experiments.

### SA-β-gal assay

Aortic tissue and VSMC senescence were detected using an association-β-galactosidase (SA-β-gal) staining kit (C0602, Beyotime). Briefly, aortic tissue and VSMCs were washed with PBS three times and fixed at room temperature for 15 min. Subsequently, they were incubated with SA-β-gal solution at 37℃ overnight. SA-β-gal positive cells that stained blue were randomly imaged in different fields of view. The percentage of senescent VSMCs was calculated as the ratio of SA-β-gal-positive cells to total VSMCs. The stained intact aortas were photographed using a digital camera, embedded in optimal cutting temperature compound and cut into 5 μm frozen sections. These sections were then photographed in a random field of view by a microscope, and the senescence of aortic wall assessed by the proportion of SA-β-gal positive areas to whole aortic wall.

### DHE staining

To evaluate ROS level in abdominal aortic tissue, DHE (dihydroethidium) staining was performed according to the protocol. Briefly, unfixed frozen sections of aortic tissue were stained with DHE (10 mM, D23107, Invitrogen) for 20 min at 37℃ in the dark and examined under a fluorescence microscope. ROS level was calculated based on red fluorescence intensity using Image J software.

### Internalization assay

To track the internalization of HMEXO and AMEXO by VSMCs, HMEXO and AMEXO were labeled with DiI. Briefly, EXO were incubated with 10 µM DiI at 37 °C for 15 min and incubation terminated with 10% FBS. After washing with PBS three times, 5 × 10^9^ DiI-HMEXO and DiI-AMEXO were added to cultured VSMCs in confocal dishes and incubated for 3 h. Finally, VSMCs were stained with anti-α-SMA (ab7817, Abcam) and DAPI. Images were obtained with a confocal fluorescence microscope (SP5-FCS; Leica).

### Exosome small RNA-sequencing

RNA from AMEXO and HMEXO was extracted using a miRNeasy Kit (217,004, Qiagen). RNA output and purity were analyzed using the Qubit® 2.0 (Q32866, Flex, Life Technologies). 20 ng of total RNA from each sample was used to build the small RNA libraries. The small RNA was sequenced using a HiSeq 2000 (genedenovo Co. Ltd, Guangzhou, China). Data were normalized using the robust multiarray analysis algorithm, and the expression of miRNAs analyzed using the edgeR algorithm for analyzing significant differences between two data sets. Heat maps and volcano maps of differential miRNAs were generated by the omicshare cloud platform (www.omicshare.com). Target gene prediction of differentially expressed miRNAs was performed with TargetScan and miRTarBase.

### Quantitative real-time PCR

Total RNA in cells, tissue, HMEXO and AMEXO was extracted using the miRNeasy Kit (217,004, Qiagen). Reverse transcription was performed using a PrimeScript RT Reagent Kit (RR037A, Takara). RT-PCR for miRNAs or mRNA of MST4 (Mammalian sterile-20-like kinase 4) was conducted using a quantitative SYBR Green RT-PCR kit (204,243, Qiagen) according to the manufacturer’s instructions. Relative expression of miRNA and mRNA in cells was normalized using U6 or cel-miR-39 and calculated by the 2 − ΔΔCt method.

### Transfection of miR-19b-3p mimic

miR-19b-3p mimic was obtained commercially from RiboBio Co., Ltd. MSCs and VSMCs were transfected with miR-19b-3p mimic or miR-19b-3p inhibitors using Lipofectamine 2000 transfection reagent (11,668,027, Invitrogen) according to the manufacturer’s protocol. Next, MSCs were cultured for 48 h for transfection and then cell supernatant collected for miR-19b-3p-AMEXO isolation.

### Lentiviral infection of MST4

The lentiviral plasmid for MST4 was purchased from TranSheepBio (Shanghai, China). The lentivirus was packaged as per the manufacturer’s protocol [29]. For stable transduction of MST4, VSMCs at 80% confluence were infected with MST4 lentivirus at a multiplicity of infection (MOI) of 10. Infection efficiency was detected by the protein level of MST4 in VSMCs using Western blotting.

### Luciferase assay

The 3’-UTR of human MST4 containing the miR-19b-3p putative target site or the mutation in the seed region of the miR-19b-3p binding site was inserted into the pmirGLO luciferase reporter vector. 293T cells were co-transfected with wild-type pmirGLO-MST4-3’-UTR or mutant pmirGLO-MST4-3’-UTR and a scrambled miRNA control or miR-19b-3p mimic by Lipofectamine 2000 (11,668,027, Invitrogen). Luciferase activity was measured 48 h after transfection using a dual luciferase reporter gene assay system (E1910, Promega).

### Western blotting

Total protein of aortic tissue, HMEXO, AMEXO or VSMCs with different treatments was extracted using a protein extraction kit (BB-3101, Bestbio) and the concentration measured by BCA assay kit (231,227, Thermo). 30 µg of protein was denatured in a loading buffer at 100 °C for 10 min. Proteins were separated by 10% SDS-PAGE and transferred to a PVDF membrane (Immobilon-P, Millipore). Subsequently, membranes were blocked with 5% skimmed milk at room temperature for 1 h and incubated at 4˚C overnight with the following antibodies: anti-CD63 (ab134045, Abcam), anti-CD81 (ab109201, Abcam), anti-TSG101 (ab125011, Abcam), Calnexin (10427-2-AP, Proteintech), anti-p16 (ab51243, Abcam), anti-p21 (ab109199, Abcam), anti-p-Drp1 (ser616) (3455, CST), anti-Drp1 (14,647, CST), anti-p-ERK1/2 (9101, CST), anti-ERK1/2 (4695, CST), anti-MST4 (3822, CST) and anti-GAPDH (2118, CST). The membranes were then incubated with secondary antibodies (7076; 7074, CST) for 1 h at room temperature and exposed in a darkroom. Quantitative analysis of Western blotting was performed using ImageJ software.

### Cell growth assay

The growth and viability of VSMCs were evaluated using a cell counting kit 8 assay kit (CK04, Dojindo). VSMCs were seeded into 96-well cell culture plates at a density of 2000 cells per well. To quantify the number of cells, cell counting kit 8 assay was performed at 0, 3, 6, 12, 24, 48 and 72 h with absorbance measured at 450 nm.

### MitoTracker staining

Mitochondrial morphology of VSMCs was determined using MitoTracker staining. Briefly, VSMCs were cultured in a 6-well plate with cover slides and then subjected to different treatments. After they reached 70–80% confluence, VSMCs were washed three times and incubated in complete DMEM medium with 25 nM MitoTracker Green FM (M7514, Invitrogen) and Hoechst 33,342 (H3570, Gibco) for 15 min. Finally, VSMCs were washed three times with DMEM medium and imaged by laser confocal microscopy. Mitochondrial fragmentation was analyzed as previously reported [30]. Briefly, six fields were randomly captured and at least 300 cells per treatment group were counted. The percentage of fragmented mitochondria among the total number of cells was calculated.

### Mito-sox staining

The level of mitochondrial ROS (mtROS) in VSMCs was measured by Mito-sox staining according to the manufacturer’s instructions. Briefly, VSMCs were cultured in a 12-well culture plate and exposed to different treatments. After washing with PBS three times, VSMCs were incubated in HBSS (14,025,092, Gibco) containing 5 µM Mito-sox (M36008, Invitrogen) at 37 °C in the dark for 15 min. Finally, VSMCs were washed with PBS and a random field of view of each sample photographed under a laser confocal microscope. The intensity of Mito-sox fluorescence was assessed by Image J software.

### Immunocytochemistry

VSMCs with different treatments were fixed with 4% paraformaldehyde (AR1069, BOSTER Biological Technology) at room temperature for 15 min. Next, fixed cells were permeabilized with 0.1% Triton X-100 in PBS solution containing 1% BSA for 45 min and then incubated with α-SMA (ab7817, Abcam) and anti-Ki67 overnight at 4 °C. Subsequently, cells were washed with PBS and incubated with secondary antibodies conjugated with fluorophores for 1 h at room temperature. Finally, cell nuclei were stained with DAPI. Isotype IgG (5415, CST) was used as a negative control. Images were taken by a fluorescence microscope. The percentage of Ki67 positive cells was calculated as the ratio of Ki67-positive cells to DAPI-positive cells ×100%.

### Statistical analysis

Statistical analyses were performed for 3 biological replicates or 6 independent experimental replicates. Normality was checked by Shapiro-Wilk and Brown-Forsythe tests, respectively. Data are expressed as mean (standard error of the mean, SEM) or median (interquartile range, IQR) where appropriate. Statistical analyses were performed using GraphPad Prism version 9.3.0. For 2 group comparisons, all data passed homogeneity tests (F tests, *P* > 0.10). Comparisons between 2 groups were determined by the 2-tailed Student *t* test for normally distributed data or Mann-Whitney *U* test for non-normal distribution. For multiple group comparisons, all data passed normality and homogeneity tests (Brown-Forsythe tests, *P* > 0.05). Comparisons among 3 or more groups were determined by 1-ANOVA analysis followed by Holm-Sidak multiple comparisons test for normal distribution. The following data passed normality and were analyzed using 2-way ANOVA followed by Tukey’s multiple comparisons tests: qRT-PCR of miR-133a-3p, miR-19b-3p and miR-30c-5p in HMEXO or AMEXO. For survival curve analysis, we used a Log-rank (Mantel-Cox) test. CCK-8 analysis was performed using a linear mixed effects model. Test results in this study are shown in Supplementary Table 2. A value of *P* < 0.05 was considered statistically significant.

## Results

### Isolation and characterization of HMEXO and AMEXO

HMEXO and AMEXO were isolated from the CDPF medium of ADMSCs at passage 3–4 from healthy donors and AAA patients, respectively. HMEXO and AMEXO were characterized by nanoparticle tracking analysis, transmission electron microscopy and Western blotting. Nanoparticle tracking analysis showed that the size distribution of most HMEXO and AMEXO ranged from 50 to 150 nm (Figure S1A). The total particle number of HMEXO was 5.71 × 10^10^ ± 3.22 × 10^7^ /mL and of AMEXO 5.96 × 10^10^ ± 4.73 × 10^7^ /mL as determined by Nanosight. There was no significant difference between HMEXO and AMEXO in terms of concentration or particle size (Figure S1B). Transmission electron microscopy showed that both HMEXO and AMEXO had a spheroid morphology (Figure S1C). Western blotting showed that HMEXO and AMEXO expressed exosomal surface markers CD81, CD63 and TSG101, and negatively expressed calnexin (Figure S1D). These data show that HMEXO and AMEXO had been successfully isolated.

### AMEXO and HMEXO treatment attenuates Ang II-induced AAA formation and aortic senescence

**Fig. 1:**
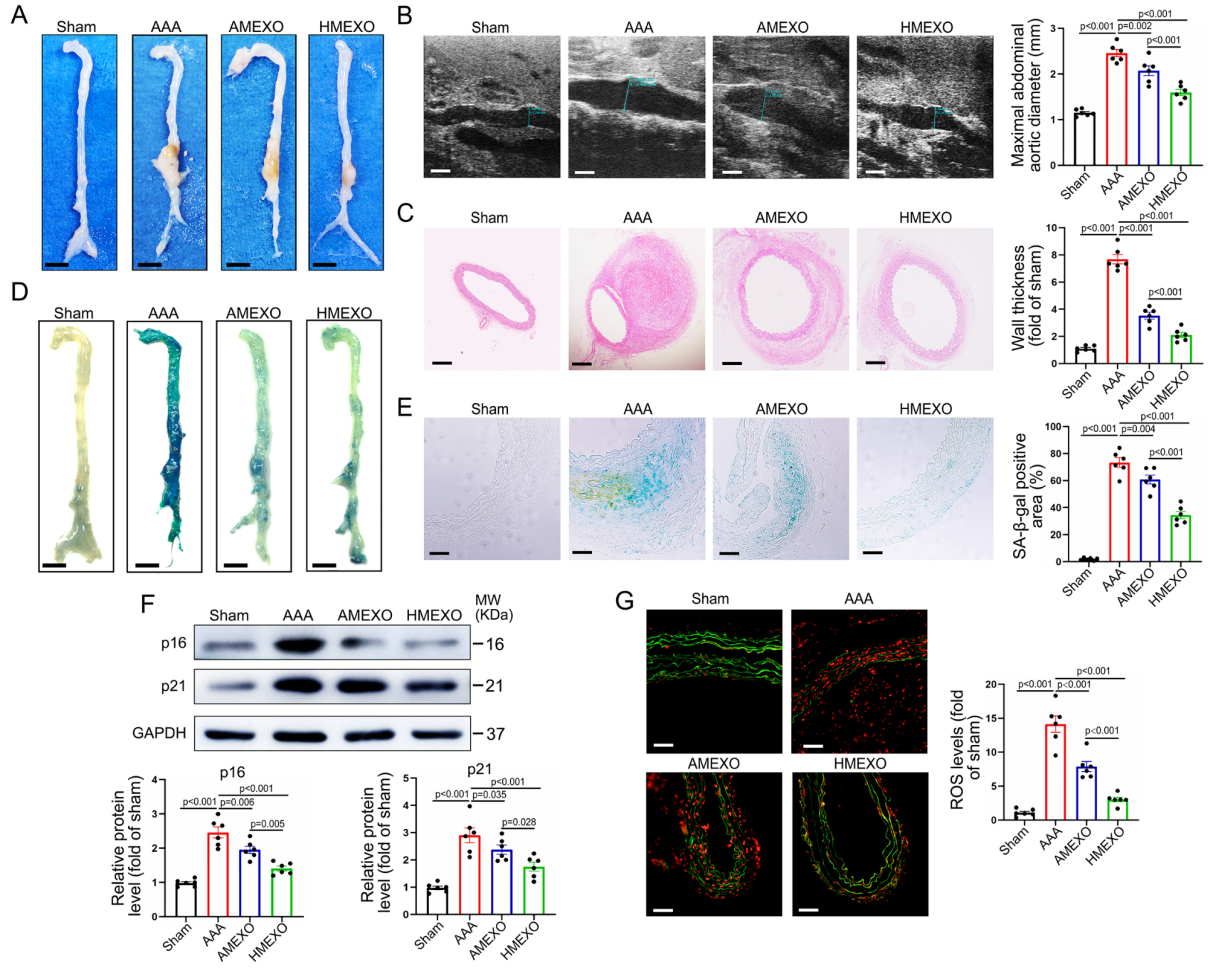
AMEXO and HMEXO treatment attenuates Ang II-induced AAA formation and aortic senescence. (A) Representative photographs of the whole aorta from control, Ang II-induced AAA mice and AAA mice treated with AMEXO or HMEXO. Scale bar: 2 mm. (B) Representative photographs of two-dimensional ultrasound and analysis of the maximum diameter of the abdominal aorta from control, Ang II-induced AAA mice and AAA mice treated with AMEXO or HMEXO (n = 6 mice). Scale bar: 1 mm. (C) Representative images of HE staining and analysis of the thickness of abdominal aortic walls from control, Ang II-induced AAA mice and AAA mice treated with AMEXO or HMEXO (n = 6 mice). Scale bar: 150 μm. (D) Representative images of SA-β-gal staining of the whole aorta from control, Ang II-induced AAA mice and AAA mice treated with AMEXO or HMEXO. Scale bar: 2 mm. (E) Representative images of abdominal aortic sections of SA-β-gal staining and analysis of the percentage of SA-β-gal staining positive areas from control, Ang II-induced AAA mice and AAA mice treated with AMEXO or HMEXO (n = 6 mice). Scale bar: 50 μm. (F) Western blotting analysis of the protein level of p16 and p21 in the aorta from control, Ang II-induced AAA mice and AAA mice treated with AMEXO or HMEXO (n = 6 mice). (G) ROS level represented by DHE fluorescence intensity was analyzed. Red fluorescence indicates superoxide and green fluorescence indicates laminae (n = 6 mice). Scale bar: 50 μm. Data are expressed as mean ± SEM. One-way ANOVA followed by Holm-Sidak multiple comparison test

To investigate the protective effects of HMEXO and AMEXO on AAA formation, Ang II-induced AAA mice were intravenously injected with HMEXO or AMEXO every 3 days starting the day following Ang II infusion, and aortic tissue collected on day 28 post Ang II infusion (Figure S2). Immunofluorescence images confirmed that DiI-HMEXO and DiI-AMEXO were present in the aortic wall (Figure S3), suggesting that HMEXO and AMEXO were effectively taken-up by the aortic wall. Notably, there was no significant difference in recruitment of HMEXO or AMEXO in the aortic wall (Figure S3). Compared with the Sham group, mortality rate of mice was significantly increased due to aortic dissection within 4 weeks of Ang II infusion, and HMEXO and AMEXO could not ameliorate mortality in Ang II-induced AAA mice (Figure S4). After Ang II infusion, the abdominal aorta of mice exhibited obvious dilation, whereas no mouse showed evidence of AAA following saline infusion (Fig. 1A). Compared with the AAA group, the dilation of the abdominal aorta was significantly reduced in the HMEXO and AMEXO groups, and further reduced in the HMEXO group (Fig. 1A). Ultrasound results also demonstrated that both HMEXO and AMEXO treatment reduced the maximum diameter of Ang II-induced AAA and HMEXO displayed a better protective effect against AAA formation (Fig. 1B). Moreover, HMEXO and AMEXO treatment reduced Ang II-induced aortic wall thickening, with a larger reduction of aortic wall thickness in HMEXO-treated mice (Fig. 1C).

**Fig. 2:**
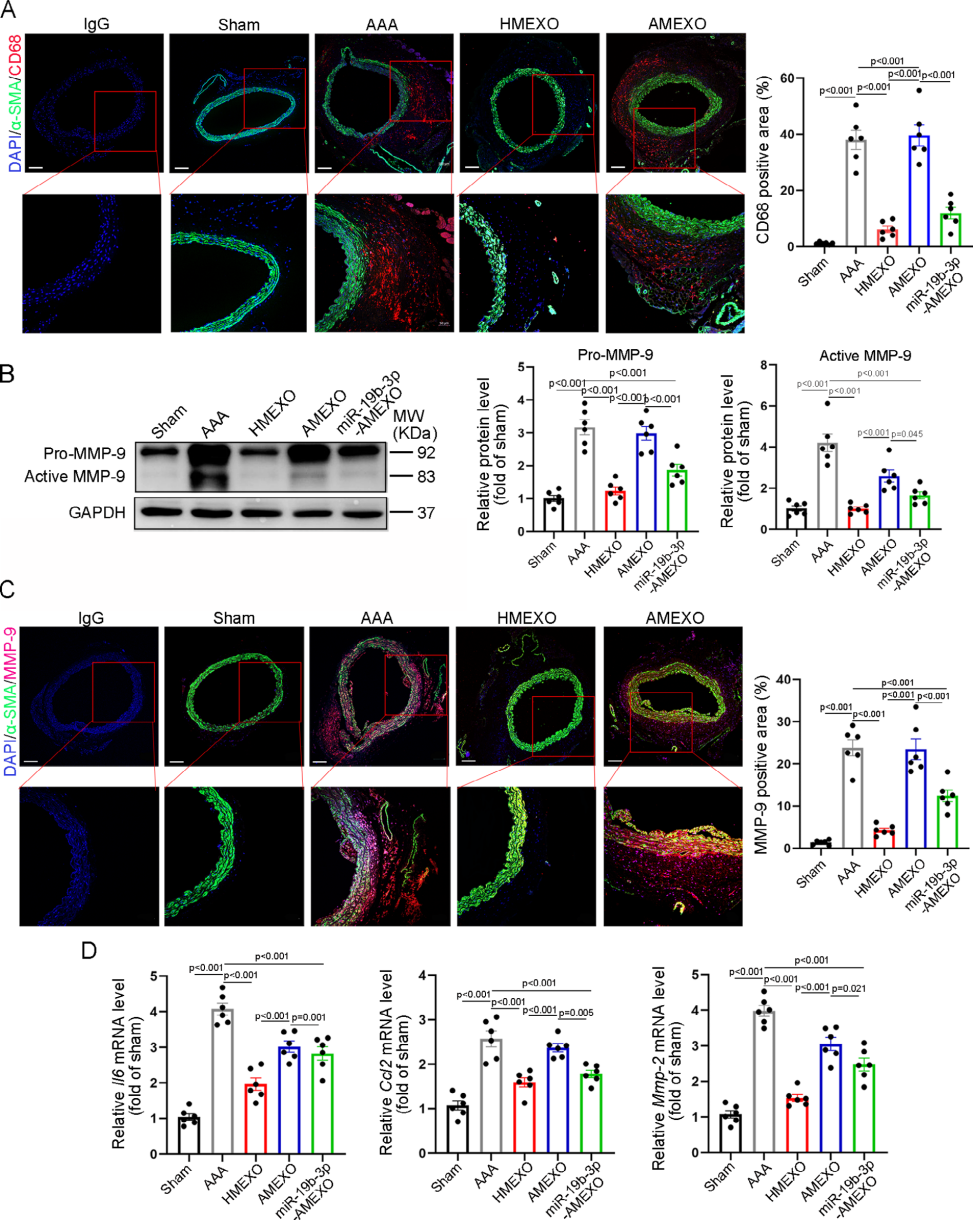
HMEXO more effectively reduced aortic inflammation than AMEXO. (A) CD68 and α-SMA immunofluorescence staining of aortic tissue sections of Sham, AAA, HMEXO, AMEXO and miR-19b-3p group (n = 6 mice). Scale bar: 100 μm. Data are expressed as mean ± SEM. (B) Western blotting and quantitative analysis of the expression of pro-MMP-9 and active MMP-9 of aortic tissue sections of Sham, AAA, HMEXO, AMEXO and miR-19b-3p group (n = 6 mice). (C) MMP-9 and α-SMA immunofluorescence staining of aortic tissue sections of Sham, AAA, HMEXO, AMEXO and miR-19b-3p group (n = 6 mice). Scale bar: 10 μm. (D) qRT-PCR analysis of Il6, Ccl2 and Mmp-2 mRNA expression of aortic tissue sections of Sham, AAA, HMEXO, AMEXO and miR-19b-3p group (n = 6 mice). Data are expressed as mean ± SEM. One-way ANOVA followed by Holm-Sidak multiple comparison test

It has been reported that VSMC senescence plays a critical role in AAA formation [7]. We examined the protective effects of HMEXO and AMEO on VSMC senescence in AAA. In the sham group, few SA-β-gal positive staining areas in the aorta were observed. Ang II infusion significantly increased the area of SA-β-gal positive staining in the whole aorta of mice, and both HMEXO and AMEXO treatment greatly reduced the SA-β-gal positive staining area in the aorta of AAA mice (Fig. 1D). Notably, sections of the abdominal aorta showed that Ang II-induced aortic senescence was mainly located in the medial region. Both HMEXO and AMEXO reduced the proportion of SA-β-gal positive staining in the abdominal aortic wall, to a greater level with HMEXO than with AMEXO treatment (Fig. 1E). We next examined the protein level of senescence-associated p16 and p21 that were significantly increased in the AAA group but greatly decreased in the HMEXO and AMEXO group. Notably, p16 and p21 protein expression was much lower in the HMEXO group than in the AMEXO group (Fig. 1F). Similarly, Ang II infusion significantly increased ROS generation in the aorta. HMEXO and AMEXO treatment dramatically downregulated the accumulation of ROS in the aorta, more so following HMEXO than AMEXO treatment (Fig. 1G). These results demonstrated that both HMEXO and AMEXO attenuated Ang II-induced AAA formation and VSMC senescence in mice, and HMEXO showed better protective effects.

Vascular inflammation has been documented as an important contributor to AAA formation [9]. Therefore, we examined MMP-9 expression and CD68-positive macrophage accumulation in the aortic wall. Compared with the sham group, the area of CD68-positive macrophages was greatly increased in the adventitia and media of the aorta in the AAA group but greatly decreased in the HMEXO and AMEXO-treated groups (Fig. 2A). More importantly, the area of CD68-positive macrophages in the medial aortic wall in the HMEXO group was greatly reduced compared with that of the AMEXO group (Fig. 2A). Pro-MMP-9 and active MMP-9 in aortic tissue of AAA mice were also more significantly reduced by HMEXO, compared with AMEXO (Fig. 2B). A similar result was obtained for MMP-9 expression in the adventitia and media of the aorta of different groups (Fig. 2C). In addition, we found that the mRNA level of pro-inflammatory factors *Il6*, *Ccl2* and *Mmp-2* in the aorta of mice was significantly downregulated by HMEXO, compared with AMEXO (Figure A similar result was obtained for MMP-9 expression in the adventitia and media of the aorta of different groups (Fig. 2D), indicating that HMEXO is more effective in reducing aortic inflammation in AAA mice than AMEXO.

### AMEXO and HMEXO treatment attenuates Ang II-induced VSMC senescence

**Fig. 3:**
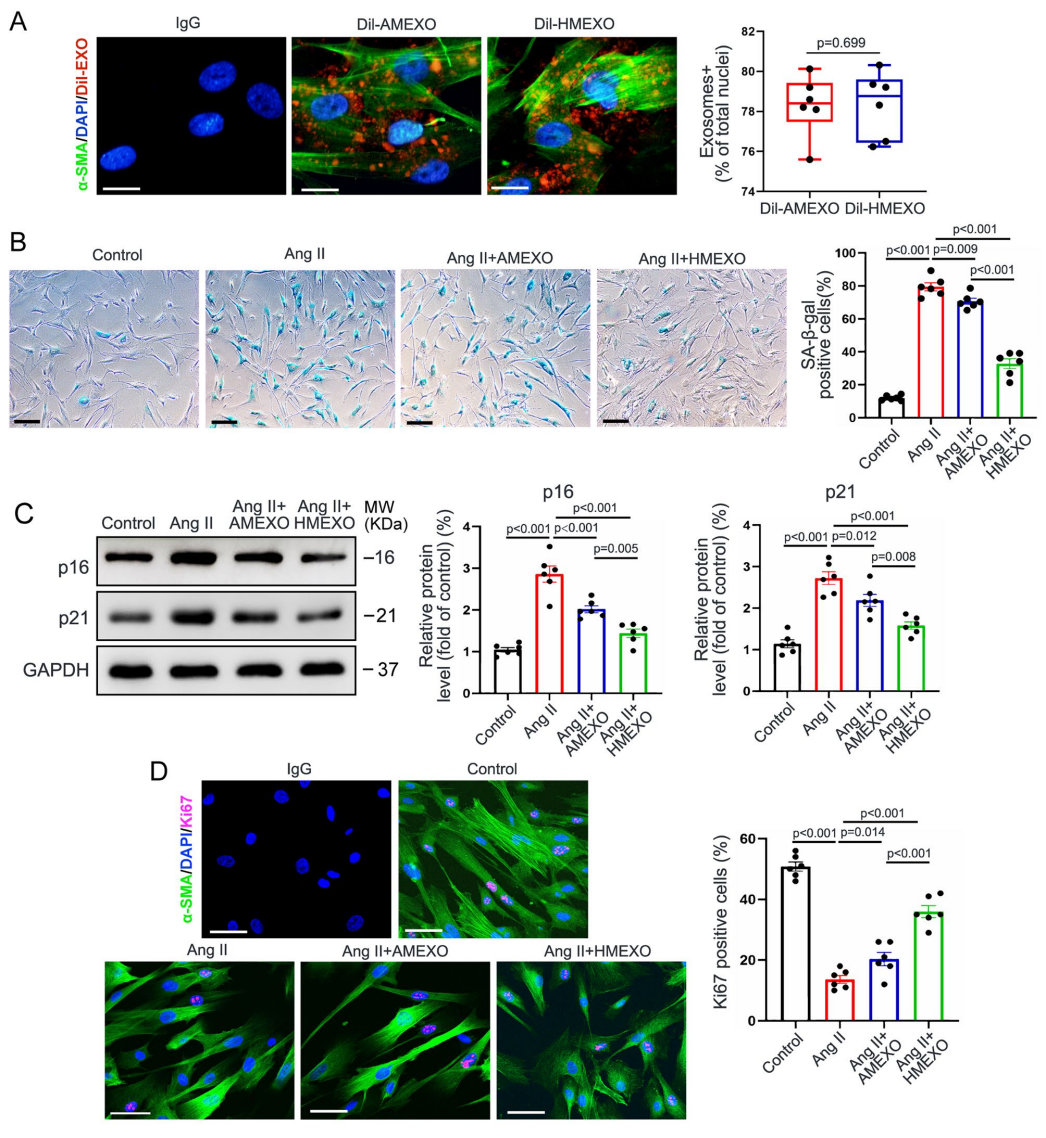
AMEXO and HMEXO treatment attenuates Ang II-induced VSMCs senescence. (A) Fluorescence microscopy images and quantitative analysis show the uptake of DiI-HMEXO and DiI-AMEXO by VSMCs (n = 6 independent experiments). Scale bar: 20 μm. (B) Representative SA-β-gal staining images and quantitative analysis of control VSMCs and VSMCs with Ang II, Ang II + HMEXO or Ang II + AMEXO treatment (n = 6 independent experiments). Scale bar: 10 μm. (C) Western blotting analysis of the protein level of p16 and p21 in control VSMCs and VSMCs with Ang II, Ang II + HMEXO or Ang II + AMEXO treatment (n = 6 independent experiments). (D) Representative immunofluorescence micrographs and quantitative analysis of Ki67 expression in control VSMCs and VSMCs with Ang II, Ang II + HMEXO or Ang II + AMEXO treatment (n = 6 independent experiments). Scale bar: 10 μm. A, Two-tailed Mann-Whitney *U* test; Data are expressed as median (IQR). B, C and D, One-way ANOVA followed by Holm-Sidak multiple comparison test; Data are expressed as mean ± SEM.

We further investigated the effect of HMEXO and AMEXO on inhibition of VSMC senescence in vitro. To determine whether HMEXO and AMEXO could be taken up by VSMCs, DiI-EXO were incubated with VSMCs for 3 h. Fluorescent microscopic imaging demonstrated that DiI-HMEXO and DiI-AMEXO were efficiently internalized by VSMCs with no significant difference in internalization efficiency between HMEXO and AMEXO (Fig. 3A). To examine whether HMEXO could attenuate Ang II-induced VSMC senescence, we treated VSMCs with 2 × 10^8^/ml, 4 × 10^8^/ml, 8 × 10^8^/ml and 1.6 × 10^9^/ml HMEXO for 48 h under 100 nM Ang II challenge. SA-β-gal staining revealed that 8 × 10^8^/ml HMEXO effectively attenuated Ang II-induced VSMC senescence, an effect that was not further increased at 1.6 × 10^9^ /ml HMEXO (Figure S5). Therefore, we used the concentration of 8 × 10^8^ /ml EXO for subsequent studies. We further investigated the effect of HMEXO and AMEXO on inhibition of VSMC senescence *in vitro.* VSMCs were treated with Ang II, Ang II + HMEXO and Ang II + AMEXO for 48 h, respectively. SA-β-gal staining showed that both HMEXO and AMEXO significantly inhibited VSMC senescence induced by Ang II, although the protective effect of AMEXO on VSMC senescence was significantly less than that of HMEXO (Fig. 3B). Western blotting revealed that HMEXO significantly reduced the protein level of p16 and p21 compared with AMEXO (Fig. 3C). To compare the effects of HMEXO and AMEXO on proliferation of senescent VSMCs, VSMCs were treated with HMEXO and AMEXO for 0, 24, 48 and 72 h. Compared with AMEXO, cell counting kit 8 assay revealed that HMEXO effectively increased proliferation of senescent VSMCs (Figure S6). Similarly, a higher proportion of Ki67-positive nuclei in senescent VSMCs was observed following HMEXO treatment (Fig. 3D), suggesting that HMEXO better promoted the proliferation of senescent VSMCs than AMEXO. These data reveal that HMEXO attenuated the senescence of VSMCs induced by Ang II, protective effects that were decreased with AMEXO.

### AMEXO and HMEXO treatment attenuates VSMC senescence by inhibiting mitochondrial fission and mtROS generation

**Fig. 4:**
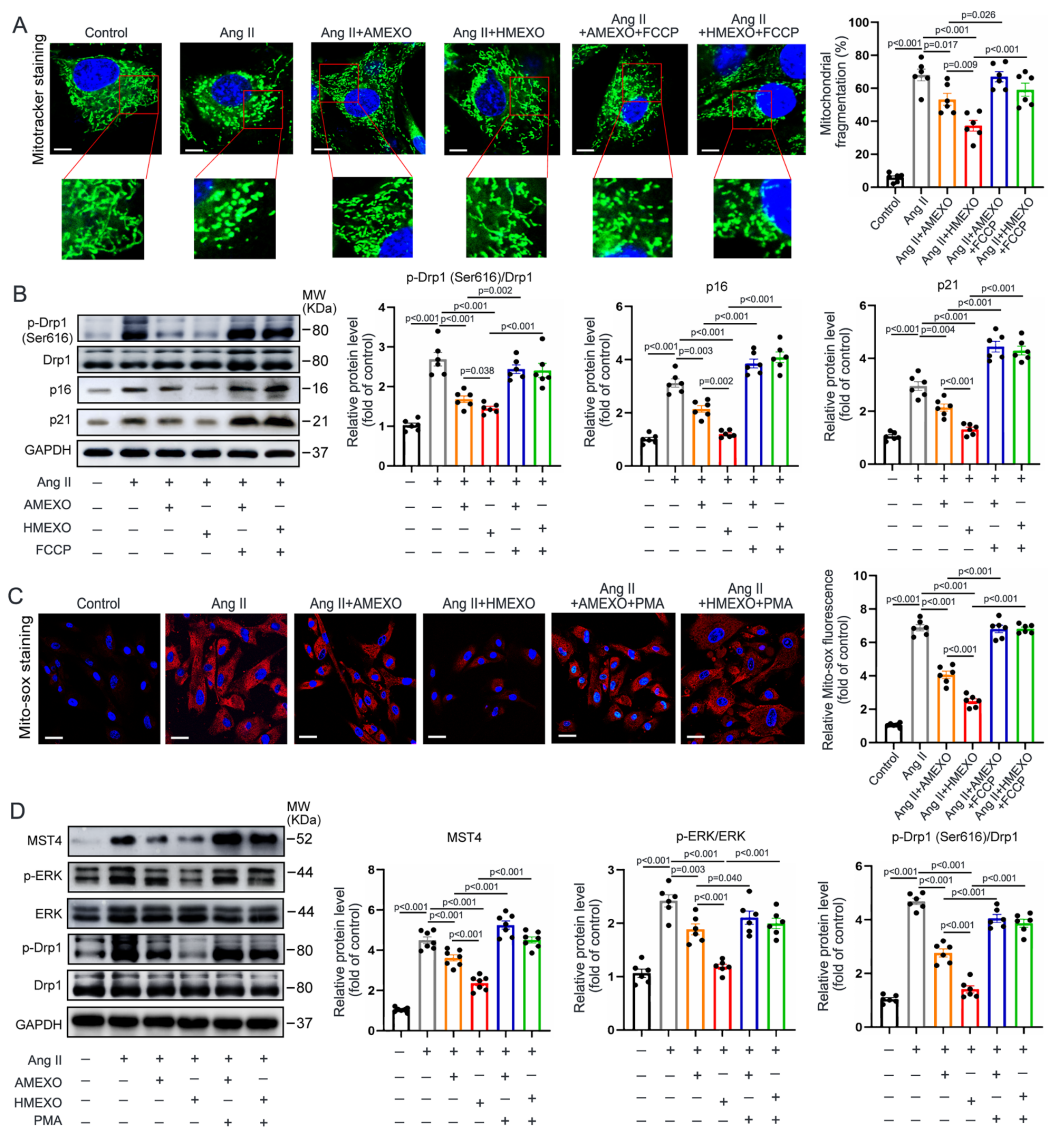
AMEXO and HMEXO treatment attenuates VSMC senescence by inhibiting mitochondrial fission and mtROS generation. (A) Representative MitoTracker staining images and quantitative analysis of control VSMCs and VSMCs following Ang II, Ang II + HMEXO, Ang II + AMEXO, Ang II + HMEXO + FCCP or Ang II + AMEXO + FCCP treatment (n = 6 independent experiments). Scale bar: 10 μm. (B) Western blotting and quantitative analysis of the protein level of p-Drp1 (Ser616), p16 and p21 in control VSMCs and VSMCs following Ang II, Ang II + HMEXO, Ang II + AMEXO, Ang II + HMEXO + FCCP or Ang II + AMEXO + FCCP treatment (n = 6 independent experiments). (C) Representative Mito-sox staining images and quantitative analysis of control VSMCs and VSMCs following Ang II, Ang II + HMEXO, Ang II + AMEXO, Ang II + HMEXO + FCCP or Ang II + AMEXO + FCCP treatment (n = 6 independent experiments). Scale bar: 20 μm. (D) Western blotting and quantitative analysis of the protein level of MST4, p-ERK and p-Drp1 (Ser616) in control VSMCs and VSMCs following Ang II, Ang II + HMEXO, Ang II + AMEXO, and Ang II + HMEXO + FCCP treatment (n = 6 independent experiments). Data are expressed as mean ± SEM. One-way ANOVA followed by Holm-Sidak multiple comparison test

Previous study has shown that mitochondrial fission and excessive ROS generation contribute to Ang II-induced VSMC senescence [12]. We explored whether HMEXO and AMEXO could inhibit cellular senescence of VSMCs by regulating mitochondrial dynamics and ROS generation. MitoTracker staining revealed that both HMEXO and AMEXO inhibited mitochondrial fission of senescent VSMCs induced by Ang II. Notably, the ability of AMEXO to inhibit mitochondrial fission was largely decreased compared with HMEXO (Fig. 4A). Western blotting showed that both HMEXO and AMEXO reduced the protein level of p-Drp1 (ser 616) and cellular senescence-associated proteins p21 and p16 in Ang II-treated VSMCs, indicating that excessive mitochondrial fission is closely associated with VSMC senescence. Moreover, compared with AMEXO, HMEXO treatment had a superior inhibitory effect on mitochondrial fission and cellular senescence of VSMCs. Remarkably, FCCP, the activator of Drp1, partially reversed the effects of HMEXO and AMEXO on inhibition of VSMC senescence (Fig. 4A, B). Mito-sox staining showed that mtROS were drastically elevated due to Ang II and decreased by both HMEXO and AMEXO treatment. Nonetheless AMEXO showed a weaker effect than HMEXO (Fig. 4C). These results suggest that compared with AMEXO, HMEXO offers superior protective effects of inhibiting VSMC senescence by regulating mitochondrial dynamics and mtROS generation.

It has been reported that Drp1 is directly activated by ERK-mediated ser616 phosphorylation [31]. Ang II was shown to accelerate vascular remodeling and vascular smooth muscle phenotypic transformation through upregulation of the HIPPO/YAP pathway, but the mechanism is unclear during aneurysm formation [32]. Importantly, MST4, an important component of this pathway, can regulate cell proliferation and transformation through activation of ERK [33]. We attempted to determine whether HMEXO and AMEXO could inhibit mitochondrial fission of senescent VSMCs via the MST4/ERK/Drp1 pathway. Western blotting analysis and qRT-PCR revealed that the protein and mRNA level of MST4 was significantly elevated in senescent VSMCs induced by Ang II (Figure S7 A, B). Consistently, the protein level of p-ERK and p-Drp1 (ser616) was increased by Ang II, suggesting that Ang II caused activation of the MST4/ERK/Drp1 pathway. Subsequently, both HMEXO and AMEXO reduced the protein level of MST4, p-ERK and p-Drp1 (ser616), although the reduction was greater following HMEXO. Remarkably, these effects of HMEXO and AMEXO were reversed by PMA, an activator of ERK (Fig. 4D). These results suggest that compared with AMEXO, HMEXO better inhibits mitochondrial fission and mtROS generation of senescent VSMCs, partly via the MST4/ERK/Drp1 pathway.

### Differential expression of miRNAs in HMEXO and AMEXO

**Fig. 5:**
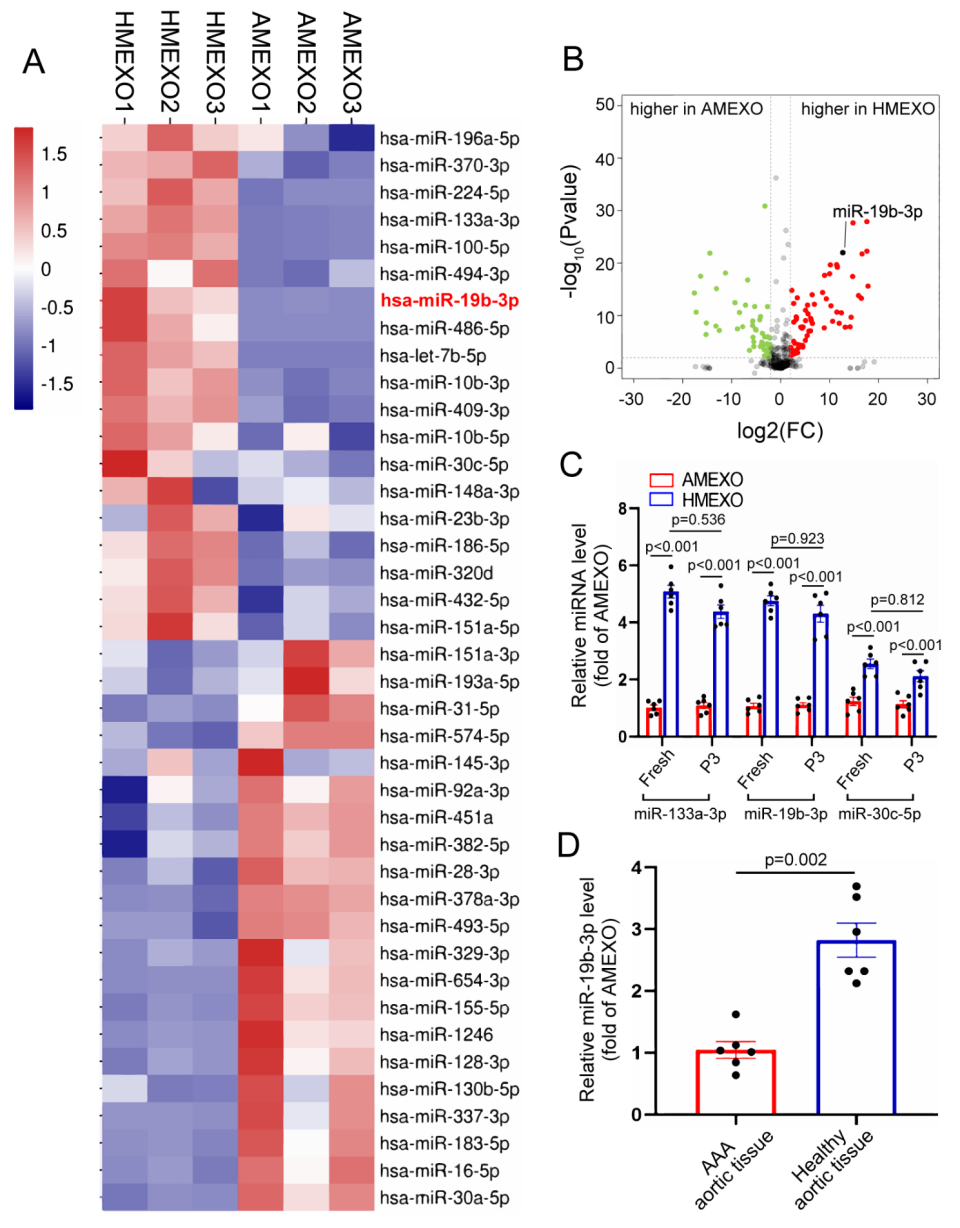
Differential expression of miRNAs in HMEXO and AMEXO. (A) Heat map showing differential expression of top 20 miRNAs between AMEXO and HMEXO. (B) Comparison of the differential relative expression of miRNAs between ANEXO and HMEXO by volcano plot. miR-19b-3p (black dot) was considered one of the significantly up-regulated miRNAs in HMEXO. (C) qRT-PCR analysis of miR-133a-3p, miR-19b-3p and miR-30c-5p expression in HMEXO or AMEXO derived from fresh and 3rd passage ADMSCs (n = 3 biological replicates). (D) qRT-PCR analysis of miR-19b-3p expression in abdominal aortic tissue of healthy donor or AAA patients (n = 3 biological replicates). Data are expressed as median (IQR). C, Two-way ANOVA followed by Tukey’s multiple comparison test. D, Two-tailed Mann-Whitney U test

To identify the exosomal miRNAs that contribute to the protective effects of HMEXO on VSMC senescence, total small RNA sequencing of HMEXO and AMEXO was performed and the corresponding miRNA expression profiles obtained. The intensity of the hybridization microarray revealed 499 miRNAs in HMEXO and AMEXO, of which 86 were differentially expressed between HMEXO and AMEXO. The heatmap shows the top 20 most highly expressed miRNAs in HMEXO and AMEXO (Fig. 5A). 48 were more highly expressed in HMEXO and 38 in AMEXO and are shown as a volcano plot (Fig. 5B). It has previously been reported that miRNAs play a pivotal role in vascular senescence [34, 35]. We examined the expression of three important miRNAs associated with vascular senescence enriched in HMEXO derived from fresh and 3rd passage ADMSCs. The results showed that no significant change in expression of these miRNAs in HMEXO and AMEXO in ADMSCs at the 3rd passage compared with fresh ADMSCs (Fig. 5C). Interestingly, hsa-miR-19b-3p has been reported to be upregulated in young adults and downregulated in older adults, [36] nevertheless whether it regulates vascular aging is unclear. In addition, the expression of miR-19b-3p was higher in healthy aortic tissue than in AAA tissue (Fig. 5D). Thus, miR-19b-3p was selected as a potential regulator of the protective effects of HMEXO on VSMC senescence.

### MSC-exosomal miR-19b-3p attenuates VSMC senescence by targeting MST4

**Fig. 6:**
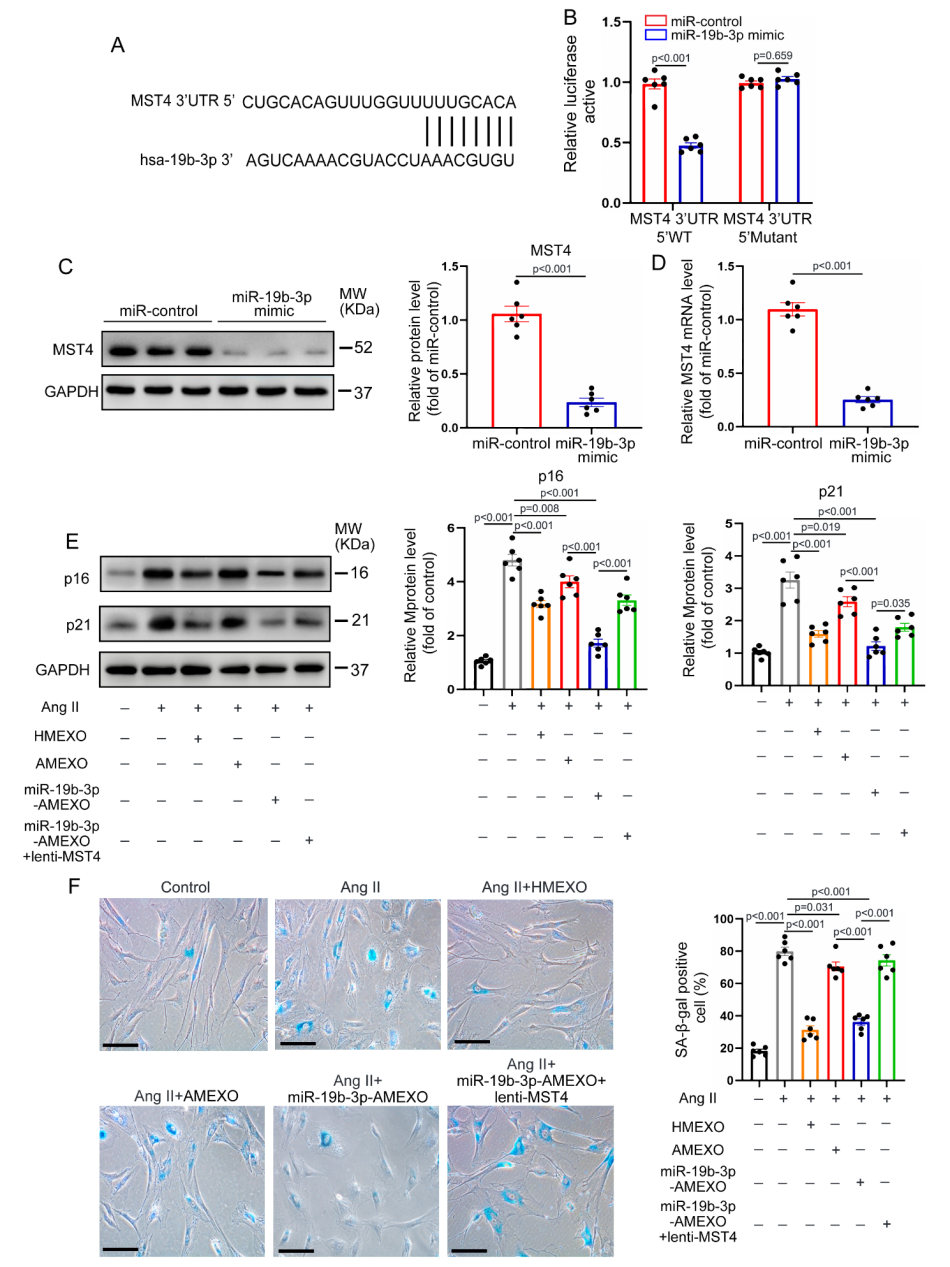
MSC-exosomal miR-19b-3p attenuates VSMC senescence by targeting MST4. (A) The potential binding sites of miR-19b-3p on the 3’UTR of MST4. (B) 293T cells were co-transfected with miR-19b-3p mimic or miRNA control and with a luciferase reporter vector containing WT or mutant 3’UTR of MST4 (n = 6 independent experiments). (C) Western blotting analysis of MST4 expression in VSMCs transfected with miR-19b-3p mimic or miR-19b-3p control (n = 6 independent experiments). (D) qRT-PCR analysis of *MST4* mRNA expression in VSMCs transfected with miR-19b-3p mimic or miR-19b-3p control (n = 6 independent experiments). (E) Western blotting and quantitative analysis of the protein level of p16 and p21 in control VSMCs and VSMCs following Ang II, Ang II + HMEXO, Ang II + AMEXO, Ang II + miR-19b-3p-AMEXO or Ang II + miR-19b-3p-AMEXO + lenti-MST4 treatment (n = 6 independent experiments). (F) Representative SA-β-gal staining images and quantitative analysis of control VSMCs and VSMCs following Ang II, Ang II + HMEXO, Ang II + AMEXO, Ang II + miR-19b-3p-AMEXO or Ang II + miR-19b-3p-AMEXO + lenti-MST4 treatment (n = 6 independent experiments). Scale bar: 10 μm. Data are expressed as mean ± SEM. B, Two-way ANOVA followed by Holm-Sidak multiple comparison test. C and D, Two-tailed Student *t* test. E and F, One-way ANOVA followed by Holm-Sidak multiple comparison test

To determine whether miR-19b-3p regulates VSMC senescence by targeting the MST4 signaling pathway, we predicted the target genes of miR-19b-3p. miRNA target prediction analysis confirmed that the miR-19b-3p binding site was within the 3’ untranslated regions (3’UTR) of MST4 (Fig. 6A). Subsequently, a dual-luciferase reporter gene assay demonstrated that miR-19b-3p significantly reduced luciferase activity of the MST4 wild-type (WT) reporter gene but had no impact on that of the MST4 mutant reporter gene (Fig. 6B). Next, VSMCs were transfected with miR-19b-3p control or mimic. Western blotting and qRT-PCR showed that miR-19b-3p mimic greatly reduced the protein and mRNA level of MST4 of VSMCs, compared with miR-19b-3p control (Fig. 6C, D). Western blotting analysis showed that the expression of MST4 was significantly decreased by miR-19b-3p mimic in mice VSMCs (Figure S8). These results demonstrated that MST4 was a target gene for miR-19b-3p.

To confirm whether deficiency of miR-19b-3p affected the anti-aging effects of AMEXO on senescence of VSMCs, we upregulated the expression of miR-19b-3p in ADMSCs derived from AAA patients with miR-19b-3p mimic, and miR-19b-3p-AMEXO were extracted. qRT-PCR showed that compared with AMEXO, the level of miR-19b-3p was significantly increased in miR-19b-3p-AMEXO (Figure S9). To determine whether the effect of MST4 overexpression could reverse the protective effect of miR-19b-3p-AMEXO on senescent VSMCs, we overexpressed MST4 in VSMCs by lentiviral transfection. The protein level of MST4 was significantly increased in VSMCs after transfection (Figure S10A). More importantly, overexpression of MST4 in VSMCs greatly enhanced cellular senescence as evidenced by the increased percentage of SA-β-gal-positive VSMCs (Figure S10B), indicating that MST4 plays a critical role in mediating VSMC senescence. We then examined the senescence of VSMCs after different treatments. Western blotting revealed that HMEXO and miR-19b-3p-AMEXO more significantly reduced the protein level of p16 and p21 of senescent VSMCs compared with AMEXO. Nonetheless these anti-aging effects of miR-19b-3p-AMEXO were reversed by overexpressed MST4 in senescent VSMCs (Fig. 6E). Similarly, compared with AMEXO, HMEXO and miR-19b-3p-AMEXO effectively improved cell proliferation and decreased SA-β-gal activity of senescent VSMCs, whereas overexpression of MST4 abrogated these protective effects of miR-19b-3p-AMEXO (Fig. 6F & Figure S11). In addition, downregulation of miR-19b-3p expression in HMEXO significantly reduced its ability to inhibit senescence of VSMCs induced by Ang II and MST4/ERK/Drp1 pathway (Figure S12). These results suggest that upregulation of miR-19b-3p enhanced the anti-aging effect of AMEXO by targeting MST4.

### MSC-exosomal miR-19b-3p reduces mitochondrial fission and mtROS generation of senescent VSMCs via the MST4/ERK/Drp1 signaling pathway

**Fig. 7:**
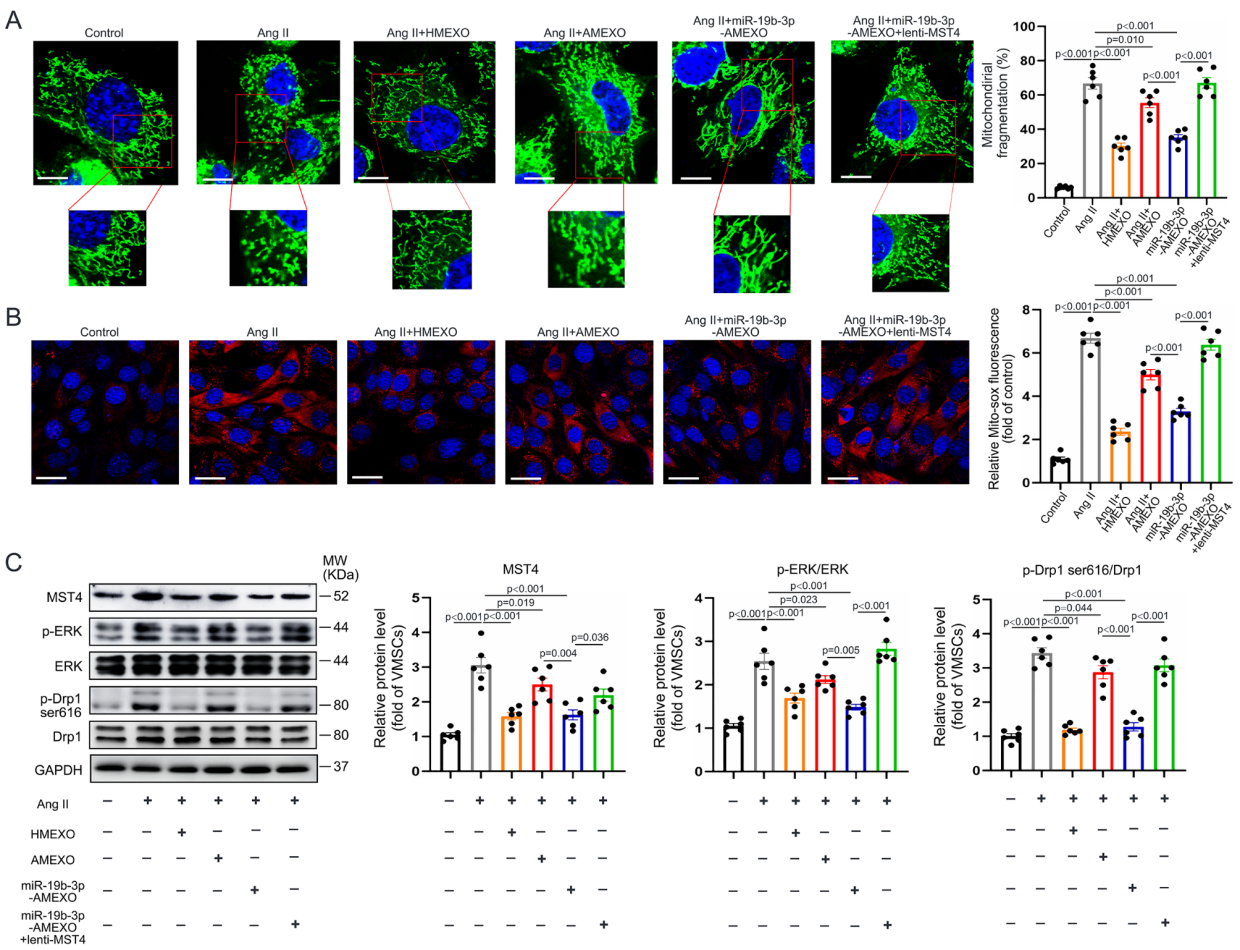
MSC-exosomal miR-19b-3p inhibits mitochondrial fission and mtROS generation in senescent VSMCs. (A) Representative MitoTracker staining images and quantitative analysis of mitochondrial length in control VSMCs and VSMCs following Ang II, Ang II + HMEXO, Ang II + AMEXO, Ang II + miR-19b-3p-AMEXO or Ang II + miR-19b-3p-AMEXO + lenti-MST4 treatment (n = 6 independent experiments). Scale bar: 10 μm. (B) Representative Mito-sox staining images and quantitative analysis of control VSMCs and VSMCs following Ang II, HMEXO, AMEXO, miR-19b-3p-AMEXO or miR-19b-3p-AMEXO + lenti-MST4 treatment (n = 6 independent experiments). Scale bar: 20 μm. (C) Western blotting and quantitative analysis of the protein level of MST4, p-ERK, and p-Drp1 (Ser616) in control VSMCs and VSMCs following Ang II, Ang II + HMEXO, Ang II + AMEXO, Ang II + miR-19b-3p-AMEXO or Ang II + miR-19b-3p-AMEXO + lenti-MST4 treatment (n = 6 independent experiments). Data are expressed as mean ± SEM. One-way ANOVA followed by Holm-Sidak multiple comparison test

To further investigate whether upregulation of miR-19b-3p in AMEXO enhanced their capacity to reduce mitochondrial fission and mtROS generation in senescent VSMCs, we examined mitochondrial morphology and mtROS level of senescent VSMCs that received HMEXO, AMEXO, miR-19b-3p-AMEXO or miR-19b-3p-AMEXO + lenti-MST4 treatment. MitoTracker staining showed that HMEXO and miR-19b-3p-AMEXO more effectively decreased mitochondrial fission of senescent VSMCs compared with AMEXO. Correspondingly, senescent VSMCs treated with HMEXO and miR-19b-3p-AMEXO displayed less mtROS generation compared with AMEXO. Nonetheless overexpression of MST4 reversed the beneficial effects of miR-19b-3p-AMEXO (Fig. 7A, B). Additionally, Western blotting showed that the protein level of MST4, p-ERK and p-Drp1 (ser616) in senescent VSMCs was more dramatically decreased by treatment with HMEXO and miR-19b-3p-AMEXO compared with AMEXO, while overexpression of MST4 partially reversed the beneficial effects of miR-19b-3p-AMEXO (Fig. 7C). These results demonstrated that MSC-exosomal miR-19b-3p regulated the mitochondrial fission and mtROS generation of senescent VSMCs by targeting the MST4/ERK/Drp1 signaling pathway.

### Overexpression of miR-19b-3p enhances the effects of AMEXO in attenuating AAA formation

To determine whether overexpression of miR-19b-3p in AMEXO could improve the therapeutic effects on AAA, Ang II-induced AAA mice were intravenously injected with AMEXO and miR-19b-3p-AMEXO every 3 days starting the day following Ang II infusion, and aortic tissue collected after 28 days (Figure S2). Immunofluorescence images revealed DiI-AMEXO and DiI-miR-19b-3p-AMEXO in the aortic wall of mice. Notably, there was no significant difference in the recruitment of AMEXO and miR-19b-3p-AMEXO in the aortic wall (Figure S3).

**Fig. 8:**
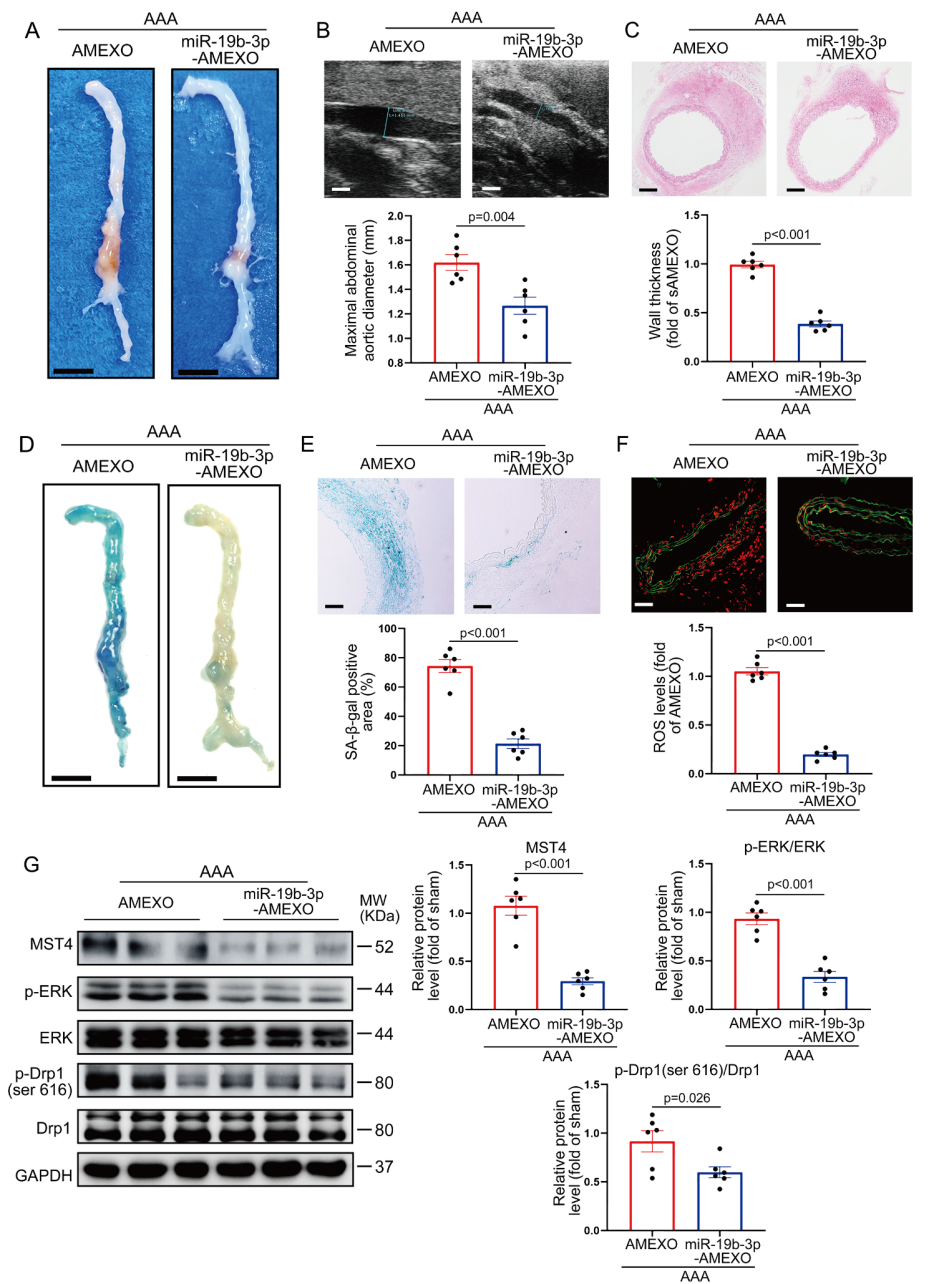
Overexpression of miR-19b-3p enhances the function of AMEXO in attenuating AAA formation and vascular senescence. (A) Representative photographs of the whole aorta from Ang II-induced AAA mice treated with AMEXO and miR-19b-3p-AMEXO. Scale bar: 2 mm. (B) Two-dimensional ultrasound imaging of abdominal aorta from Ang II-induced AAA mice treated with AMEXO and miR-19b-3p-AMEXO and analysis of the maximum aortic diameter (n = 6 independent experiments). Scale bar: 1 mm. (C) HE staining of abdominal aortic aneurysm sections from Ang II-induced AAA mice treated with AMEXO and miR-19b-3p-AMEXO and analysis of aortic thickness (n = 6 independent experiments). Scale bar: 150 μm. (D) Representative images showing SA-β-gal staining of the whole aorta from Ang II-induced AAA mice treated with AMEXO and miR-19b-3p-AMEXO. Scale bar: 2 mm. (E) Representative images of abdominal aortic sections after SA-β-gal staining from Ang II-induced AAA mice treated with AMEXO and miR-19b-3p-AMEXO. Percentage of SA-β-gal staining positive area was analyzed (n = 6 independent experiments). Scale bar: 50 μm. (F) DHE staining of abdominal aortic sections from Ang II-induced AAA mice treated with AMEXO and miR-19b-3p-AMEXO and ROS levels represented by DHE fluorescence intensity were analyzed. Red fluorescence indicates superoxide and green fluorescence indicates laminae (n = 6 independent experiments). Scale bar: 50 μm. (G) Western blotting analysis of the protein level of MST4, p-ERK and p-Drp1 (ser616) in the aorta from Ang II-induced AAA mice treated with AMEXO and miR-19b-3p-AMEXO (n = 6 independent experiments). Data are expressed as mean ± SEM. Two-tailed Student *t* test

As shown in Fig. 8A, the size of bulge in the abdominal aorta was significantly reduced in the miR-19b-3p-AMEXO group compared with the AMEXO group (Fig. 8A) although there was no significant difference in mouse mortality (Figure S13). Ultrasound imaging revealed that the maximal internal diameter of the abdominal aorta was reduced in the miR-19b-3p-AMEXO compared with the AMEXO group (Fig. 8B). Similarly, compared with the AMEXO group, aortic wall thickness as evidenced by HE staining was greatly decreased in the miR-19b-3p-AMEXO group (Fig. 8C). Further, we evaluated the effects of AMEXO and miR-19b-3p-AMEXO on inhibition of senescence of aorta in AAA mice. Similar to the results in vitro, miR-19b-3p-AMEXO more significantly reduced the proportion of SA-β-gal positive areas in the aorta of AAA mice, compared with AMEXO (Fig. 8D, E). Overexpression of miR-19b-3p enhanced the capacity of AMEXO to inhibit aortic ROS level in AAA mice (Fig. 8F). Western blotting results also showed that miR-19b-3p-AMEXO treatment significantly reduced the expression level of MST4, p-ERK and p-Drp1 (ser616) in the aortic tissue of AAA mice compared with AMEXO treatment (Fig. 8G). These results demonstrated that overexpression of miR-19b-3p significantly enhanced the ability of AMEXO to attenuate Ang II-induced AAA formation and senescence of aorta in mice.

## Discussion

In this study, we identified that HMEXO effectively inhibited Ang II-induced AAA formation and VSMC senescence, and these beneficial effects were impaired in AMEXO. Furthermore, we found that HMEXO, by regulating the MST4/ERK/Drp1 signaling pathway, ameliorated VSMC senescence through inhibition of mitochondrial fission due to the high level of miR-19b-3p. Finally, overexpression of miR-19b-3p in AMEXO improved their protective effect against Ang II-induced AAA formation and VSMC senescence.

MSCs are a promising strategy in the treatment of cardiovascular diseases [37, 38]. EXO, the major component of stem cell paracrine action and intercellular communication, have shown outstanding therapeutic anti-aging effects [39, 40]. It has been reported that umbilical cord MSC-EXO rejuvenate senescent adult bone marrow-derived MSCs, alleviate aging phenotypes and increase self-renewal capacity and telomere length [41]. Most of these MSC-EXO have been derived from young or healthy donors. Nonetheless the anti-senescent effect of AMEXO derived from AAA patients has not been investigated. Our previous study showed that ADMSCs derived from a patient with AAA exhibited senescence phenomena characterized by decreased proliferation ability and altered mitochondrial morphology [23]. Whether the contents of MSC-EXO derived from such patients are altered and whether their anti-aging properties are compromised remains to be elucidated. In this study, we found that compared with HMEXO, AMEXO failed to effectively inhibit SA-β-gal activity and the senescence-related protein level of p16 and p21 in senescent VSMCs and aortic tissue of Ang II-induced AAA mice, indicating impairment of the protective effects of AMEXO against VSMC senescence and AAA formation. Although multiple studies have shown that the physiological state of cells can impact the therapeutic capacity of EXO, [40, 42] the potential mechanisms of an impaired anti-senescent effect of AMEXO have not been elucidated.

An imbalance of mitochondrial fusion and fission is an intrinsic factor that contributes to cellular oxidative stress and senescence [43, 44]. Mitochondrial fission and ROS generation have been observed in Ang II-induced vascular senescence [12]. Consistently, we found that mitochondrial fission and mtROS were greatly increased in Ang II-induced senescent VSMCs accompanied by an increased protein level of p-Drp1 (ser616), p16 and p21. Furthermore, mitochondrial fission and mtROS level were attenuated by HMEXO treatment of senescent VSMCs, whereas AMEXO exhibited a weaker therapeutic effect. The effects of HMEXO and AMEXO on mitochondrial fission and mtROS level in senescent VSMCs were abrogated by mitochondrial fission activator FCCP. Moreover, HMEXO greatly inhibited the increased p16 and p21 protein expression and ROS level in mouse aortic tissue induced by Ang II infusion, whereas the effects were largely reduced in AMEXO-treated mice. These results suggest that HMEXO inhibited VSMCs and aorta senescence by regulating mitochondrial fission and mtROS generation, and these effects were impaired in AMEXO. Nonetheless the potential molecular mechanism of HMEXO inhibition of VSMCs and aortic senescence is unknown.

miRNAs are important components of EXO, displaying a regulatory role in inhibiting cellular senescence [45]. It has been shown that miR-221-3p in perivascular adipose tissue EXO promotes proliferation and migration of VSMCs mediating vascular remodeling [46]. miR-214 in endothelium-derived EXO inhibits vascular senescence and promotes cardiovascular generation [47]. Recent studies have shown that miR-19b-3p promotes stem cell proliferation and inhibits their senescence [48]. We analyzed the miRNA expression in HMEXO and AMEXO by miRNA sequencing. Expression of miR-19b-3p associated with vascular senescence was significantly higher in HMEXO than AMEXO derived from fresh and 3rd passage ADMSCs. Restoration of miR-19b-3p in AMEXO improved their ability to inhibit senescence of VSMCs and aortas in AAA mice and limit AAA formation. Furthermore, overexpression of miR-19b-3p in AMEXO enhanced their ability to inhibit mitochondrial fission and mtROS in senescent VSMCs. MMPs and pro-inflammatory factors in AAA mice are critical mediators of aortic aneurysm expansion [49]. Notably, miR-19b-3p-enriched HMEXO and miR-19b-3p-AMEXO more effectively suppressed the expression of MMP-9 and pro-inflammatory factors in the aorta of AAA mice, suggesting that miR-19b-3p could be a potential factor in the regulation of VSMC senescence and AAA formation. Nonetheless the potential mechanisms that underlie the mediation of AAA formation by miR-19b-3p have not been fully elucidated. It has been reported that miR-19 can target PTEN that regulates VSMCs proliferation and apoptosis to mediate AAA formation [50, 51]. Furthermore, miR-19b-3p promotes cell proliferation via interaction with lncRNA H19, [52] a known key regulator of AAA formation [53]. These findings indicate that miR-19b-3p mediates AAA formation by a complex mechanism. The ERK signaling pathway regulates mitochondrial fission and dysfunction, [54] and ERK activation promotes Drp1-dependent mitochondrial fission [31]. In addition, MST4 mediates cell proliferation by regulating the ERK pathway [33]. We also found by dual-luciferase reporter gene assay that MST4 is a direct target gene of miR-19b-3p. This information suggests that the MST4/ERK/Drp1 signaling pathway is involved in miR-19b-3p-mediated senescence and AAA formation. Furthermore, the protein level of MST4 and p-ERK was increased by Ang II treated-VSMCs and decreased by HMEXO and AMEXO. Notably, AMEXO showed a weaker therapeutic effect than HMEXO. Furthermore, PMA, an ERK activator, partly reversed the reduced protein level of MST4, p-ERK and p-Drp1 by HMEXO and AMEXO treatment. In functional recovery experiments, upregulation of miR-19b-3p in AMEXO improved the protective effect of AMEXO on inhibiting mitochondrial fission and ROS generation and reduced the protein level of MST4, p-ERK and p-Drp1 in senescent VSMCs. These effects were abrogated by overexpression of MST4. Notably, overexpression of miR-19b-3p in AMEXO enhanced their therapeutic effects in inhibiting Ang II-induced AAA formation and aortic senescence, and reduced the protein level of MST4, p-ERK and p-Drp1 in the AAA aortic tissue of mice. These results confirm that miR-19b-3p in HMEXO plays a critical role in regulating mitochondrial fission and ROS generation in senescent VSMCs to protect against AAA formation and aortic senescence by targeting the MST4/ERK/Drp1 signaling pathway.

Our study has several limitations. First, although it was established that dysregulated mitochondrial dynamics and mtROS accumulation contribute to VSMC senescence and AAA formation, it is unknown whether HMEXO and AMEXO have other effects such as regulation of telomerase shortening and chromatin abnormalities to protect VSMC senescence in aortic aneurysm. Second, the mechanism by which miR-19b-3p-enriched HMEXO suppress pro-inflammatory factor production in Ang II-induced AAA mice remains to be elucidated. Third, whether MST4 KO mice exhibit AAA inhibition should be determined by further study. Fourth, in addition to senescence, Ang II has been shown to induce both VSMC proliferation and apoptosis, thus an additional trigger in the key cell experiments needs to be determined. Finally, the participants and mice in our study were all males, limiting generalizability to the female population.

In conclusion, we found that miR-19b-3p enriched in HMEXO, partially via the MST4/ERK/Drp1 pathway, inhibited VSMC senescence and attenuated AAA formation by regulating mitochondrial fission and mtROS generation. These beneficial effects were impaired in AMEXO. Our study provides a novel target and mechanistic insight into AAA-associated VSMC senescence, paving the way for EXO as a non-cellular therapy to prevent AAA formation.

## Electronic supplementary material

Below is the link to the electronic supplementary material.

Supplementary Material 1

## Data Availability

The data that support the findings of this study are available from the corresponding author upon reasonable request. The raw small RNA sequencing data have been submitted to SRA (Sequence Read Archive) under accession number PRJNA899813.
