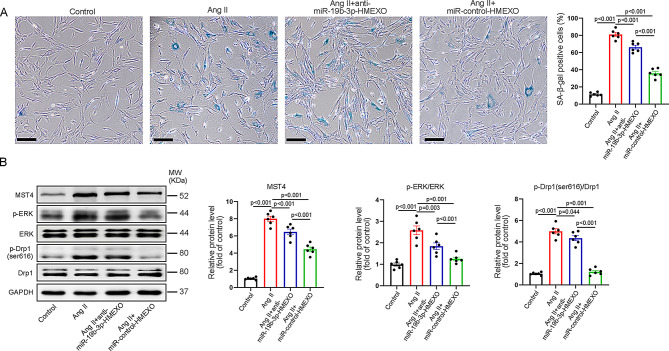# Correction: MicroRNA-19b-3p dysfunction of mesenchymal stem cell-derived exosomes from patients with abdominal aortic aneurysm impairs therapeutic efficacy

**DOI:** 10.1186/s12951-025-03490-z

**Published:** 2025-05-30

**Authors:** Yuxiao Zhang, Xiaoran Huang, Tucheng Sun, Linli Shi, Baojuan Liu, Yimei Hong, Qing-Ling Fu, Yuelin Zhang, Xin Li

**Affiliations:** 1 School of Medicine, South China University of Technology, Guangzhou, China; 2 Department of Emergency Medicine, Guangdong Provincial People’s Hospital (Guangdong Academy of Medical Sciences), Southern Medical University, Guangzhou, Guangdong China; 3 Otorhinolaryngology Hospital, The First Affiliated Hospital, Sun Yat-sen University, Guangzhou, China

**Correction: Journal of Nanobiotechnology (2023) 21:135**

10.1186/s12951-023-01894-3

In this article the wrong figure appeared as Fig. 4 and Fig. S12. The figures should have appeared as shown below.

Incorrect Fig. 4


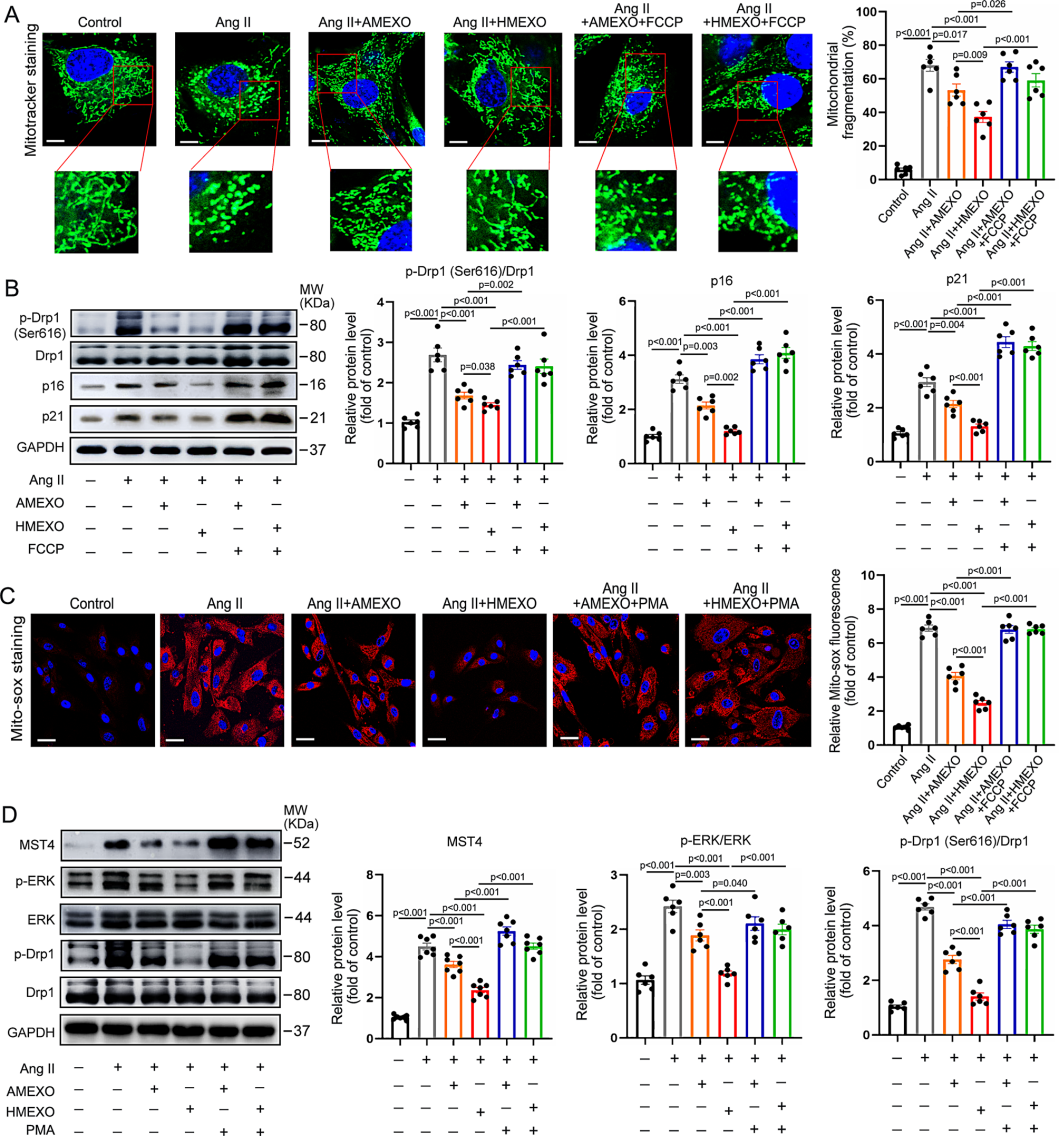


Corrected Fig. 4


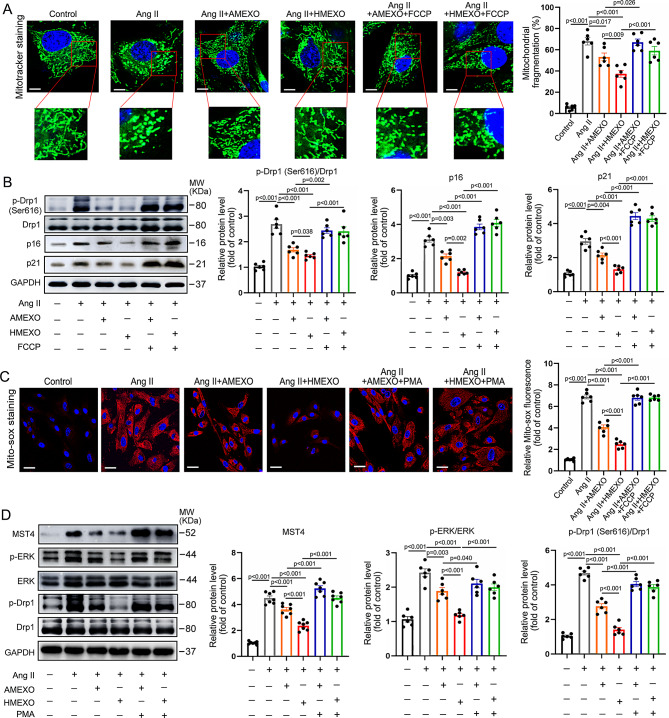


Incorrect Fig. S12


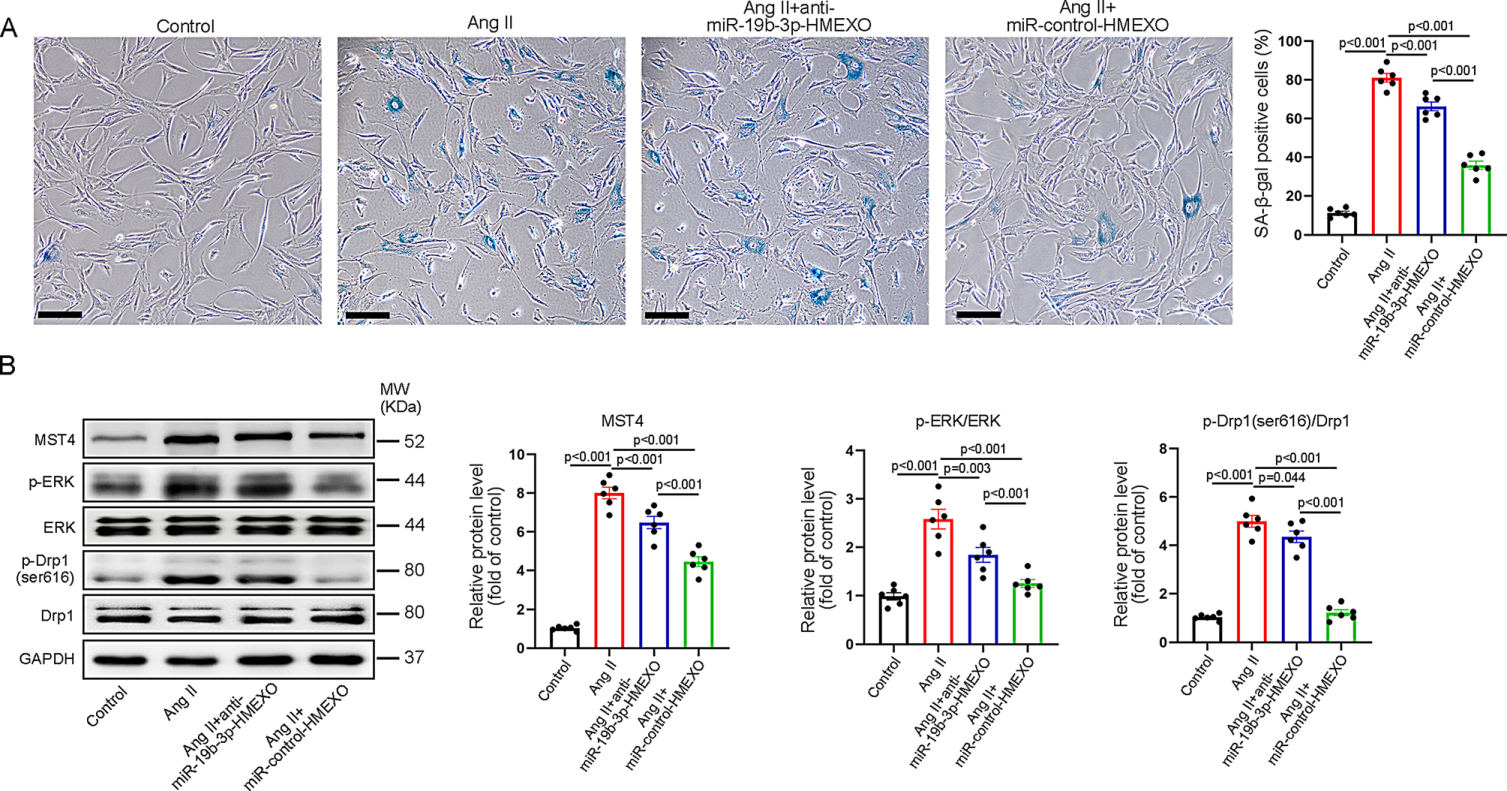


Corrected Fig. S12