# Supplementary material for: MicroRNA-19b-3p dysfunction of mesenchymal stem cell-derived exosomes from patients with abdominal aortic aneurysm impairs therapeutic efficacy
Source: J Nanobiotechnology. 2023 Apr 26;21:135. doi: 10.1186/s12951-023-01894-3 (PMC10131394; doi:10.1186/s12951-023-01894-3)
Supplement: Supplementary file 1 — Supplementary Material 1 [file 12951_2023_1894_MOESM1_ESM.docx]

**MicroRNA-19b-3p Dysfunction of Mesenchymal Stem Cell-derived Exosomes from patients with Abdominal Aortic Aneurysm Impairs Therapeutic Efficacy**

Yuxiao Zhang^1,2†^, Xiaoran Huang^2†^, Tucheng Sun^2†^, Linli Shi^2^, Baojuan Liu^2^, Yimei Hong^2^, Qing-Ling Fu^3^, Yuelin Zhang^2*^, Xin Li^1,2*^

^1^School of Medicine, South China University of Technology, Guangzhou, China;

^2^Department of Emergency Medicine, Guangdong Provincial People's Hospital, Guangdong Academy of Medical Sciences, Guangzhou, China;

^3^Otorhinolaryngology Hospital, The First Affiliated Hospital, Sun Yat-sen University, Guangzhou, China;

†Yuxiao Zhang, Xiaoran Huang and Tucheng Sun contributed equally to this work

*Address correspondence to:

Dr. Xin Li or Dr. Yuelin Zhang

Department of Emergency, Guangdong Provincial People's Hospital, Guangdong Academy of Medical Sciences, Guangzhou, Guangdong, China.

Tel. 86-20-83827812-20974

E-mail: [sylixin@scut.edu.cn](mailto:sylixin@scut.edu.cn) (Xin Li) or [zhangyuelin1999@163.com](mailto:zhangyuelin1999@163.com) (Yuelin Zhang)

**Supplementary Table 1. Demographic characteristics of the study subjects.**

| Total subjects | Control | AAA | *P* value |
| --- | --- | --- | --- |
|  | 3 | 3 | 一 |
| Age (y), median (IQR) | 63 (56, 71) | 66 (57, 75) | 0.713 |
| Male (n, %) | 3 (100%) | 3 (100%) | 一 |
| Height (cm), median (IQR) | 167 (161, 174) | 168 (160, 175) | 0.993 |
| Weight (kg), median (IQR) | 62.3 (53.5, 72.4) | 63.6 (56.1, 70.6) | 0.932 |
| BMI (kg/m2), median (IQR) | 21.124 (19.635, 23.125) | 22.368 (19.755, 24.574) | 0.838 |
| BSA (m2), median (IQR) | 1.663 (1.514, 1.836) | 1.685 (1.541, 1.818) | 0.991 |
| Smoking (n, %) | 2 (66.7%) | 2 (66.7%) | 一 |
| Hypertension (n, %) | 2 (66.7%) | 3 (100%) | 一 |
| Location of the fat biopsy | Subcutaneous | Subcutaneous | 一 |

BMI: body mass index; BSA: body surface area; AAA: abdominal aortic aneurysm; BSA=0.0061 ∗ height (cm) + 0.0128 ∗ weight (kg) – 0.1529


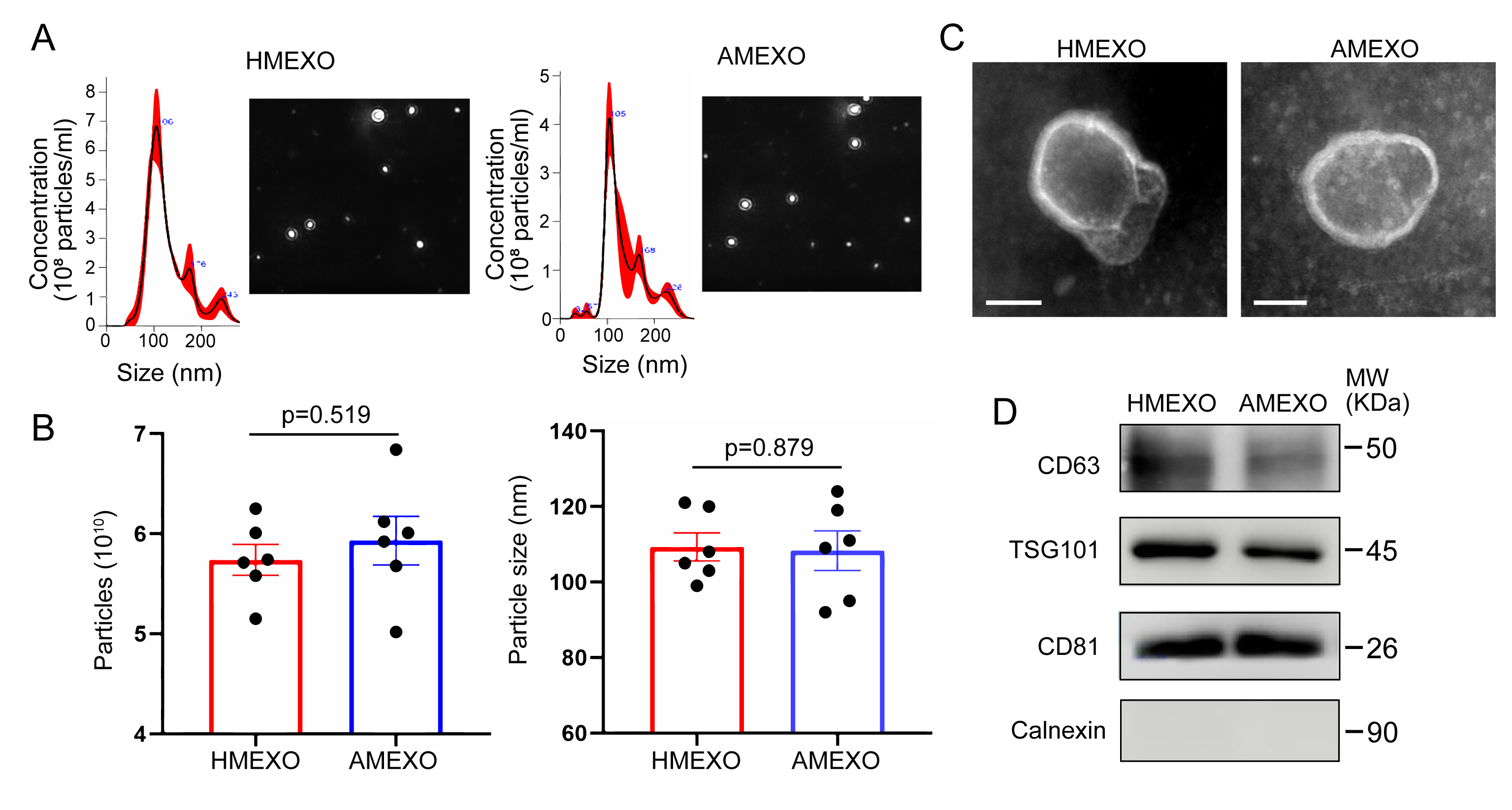


**Supplemental Figure 1. Characterization of HMEXO and HMEXO** (A) Representative results of size distribution and images of HMEXO and AMEXO. (B) Comparison of concentration and mean particle size of AMEXO and HMEXO by nanoparticle tracking analysis (n=3 biological replicates). (C) Representative results of the nano-size vesicles of HMEXO and AMEXO photographed by transmission electron microscopy. Scale bar: 50 nm. (D) Exosomal markers CD63, CD81, and TSG101 were positive and calnexin was negative in AMEXO and HMEXO on Western blotting analysis. Data are expressed as median (interquartile range, IQR). B, Two-tailed Mann-Whitney U test.


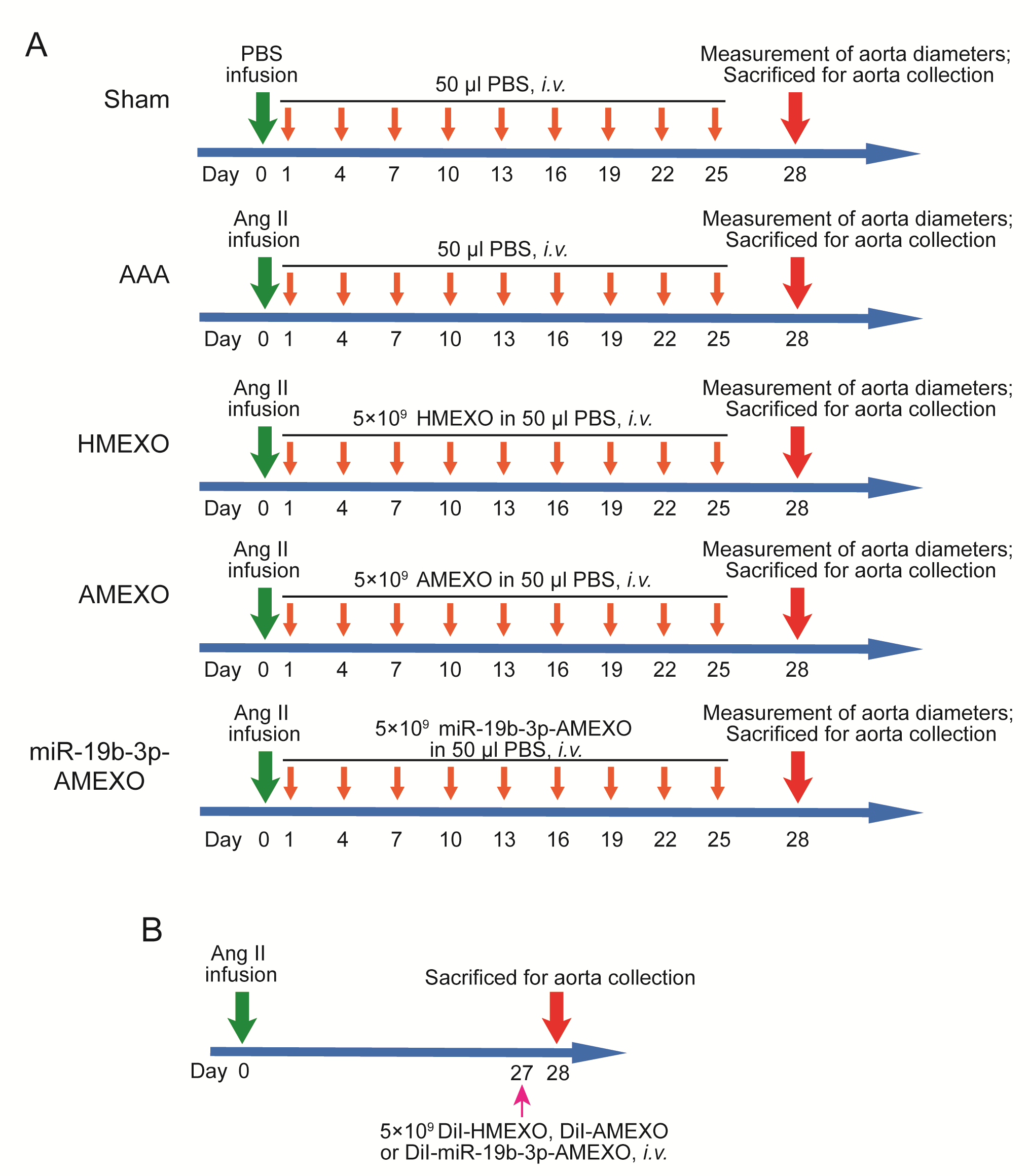


**Supplemental Figure 2. Schematic diagram of the animal study.** (A) Schematic diagram of HMEXO, AMEXO and miR-19b-3p-AMEXO treatment in Ang II-induced AAA mice. (B) Schematic diagram of study of uptake effects of AAA mice on DiI-HMEXO, DiI-AMEXO or DiI-miR-19b-3p-AMEXO.


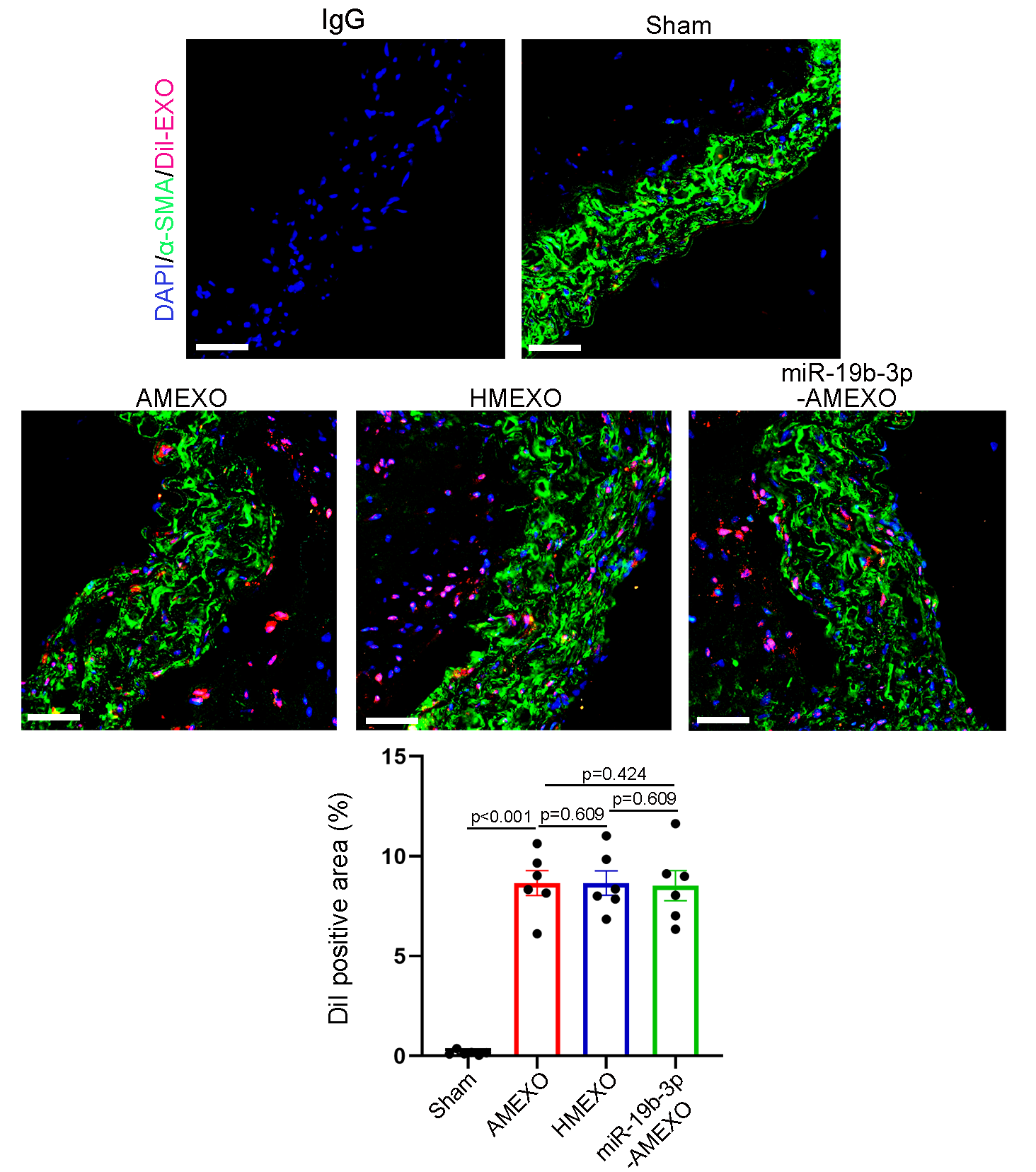


**Supplemental Figure 3. DiI-HMEXO, DiI-AMEXO and** **DiI-miR-19b-3p-AMEXO were concentrated in the abdominal aortic wall.** Representative fluorescence images of DiI-HMEXO, DiI-AMEXO, DiI-miR-19b-3p-AMEXO and α-SMA staining of abdominal aortic tissue sections of AAA mice (n=6 mice). Scale bar: 20μm. Data are expressed as mean ± SEM. One-way ANOVA followed by Holm-Sidak multiple comparison test.


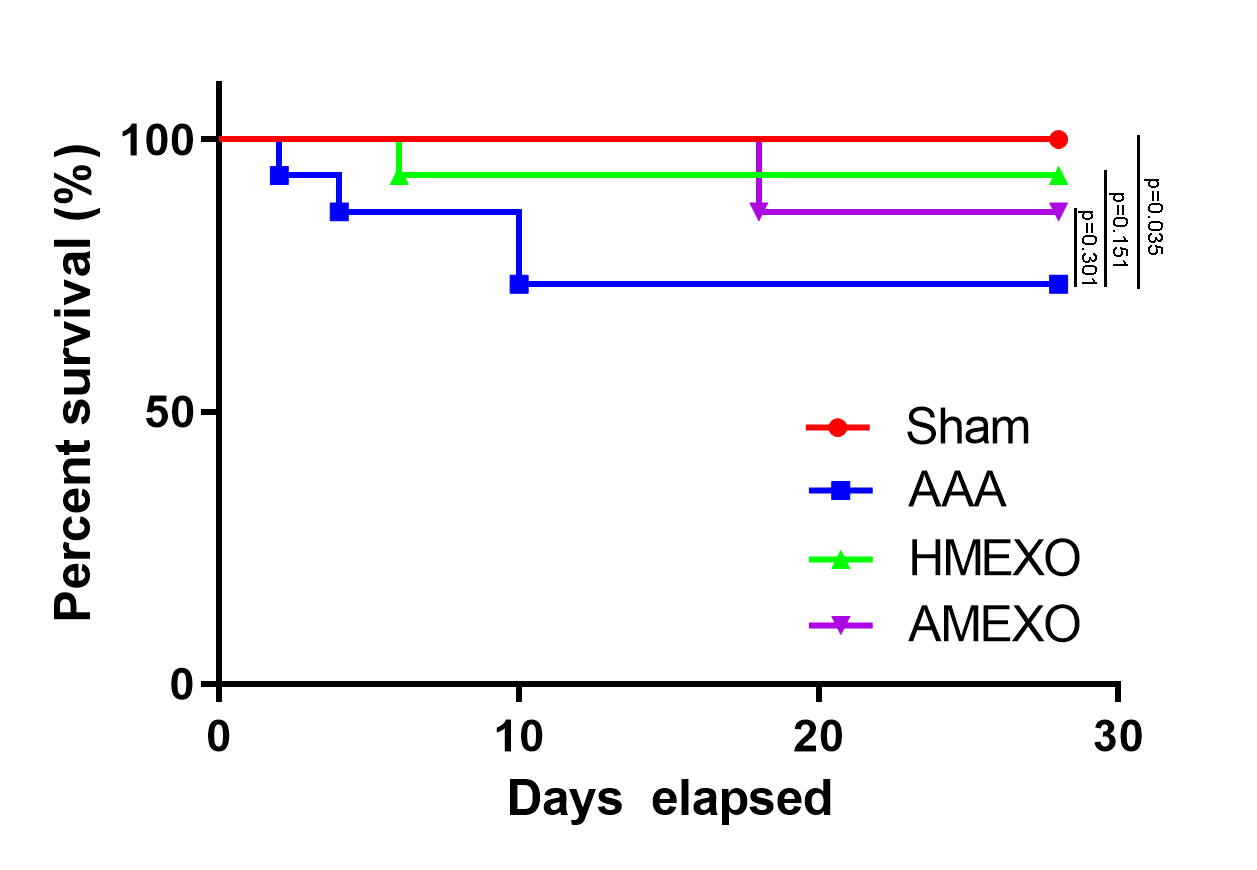


**Supplemental Figure 4. Kaplan-Meier survival curve in *ApoE^− /−^* mice and *ApoE^− /−^* mice following Ang II, AMEXO or HMEXO treatment (n=10 mice). Data are expressed as mean ± SEM. Statistical significance was determined by Log-rank (Mantel-Cox) test.**


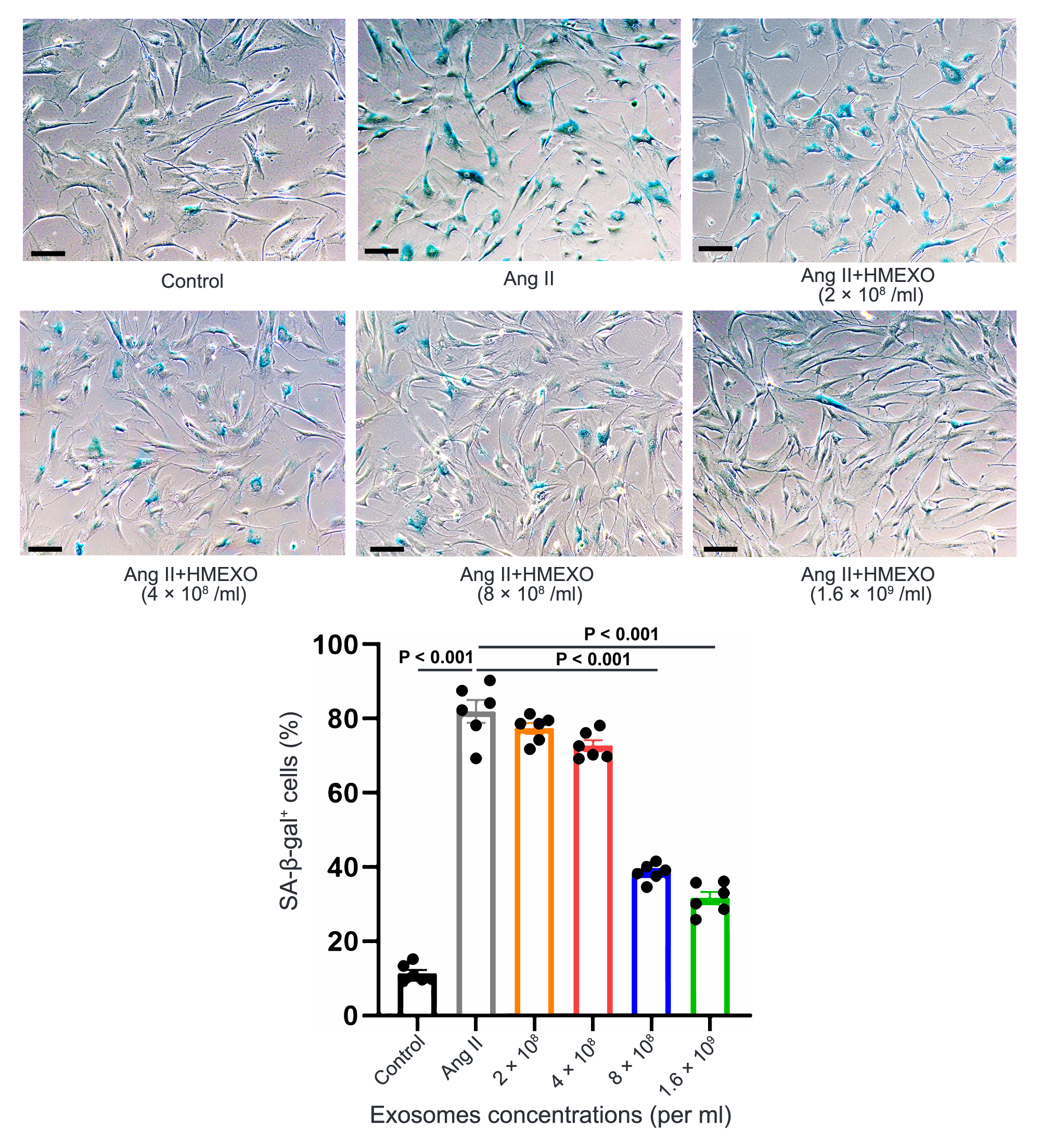


**Supplemental Figure 5. HMEXO inhibited VSMC senescence induced by Ang II in a dose-dependent manner.** Representative images and quantitative analysis of SA-β-gal staining of VSMCs treated with Ang II (100 nM) and different concentrations of HMEXO (2×10^8^ /ml, 4×10^8^ /ml, 8×10^8^ /ml and 1.6×10^9^ /ml HMEXO) (n=6 independent experiments). Scale bar: 10 μm. Data are expressed as mean ± SEM. One-way ANOVA followed by Holm-Sidak multiple comparison test.


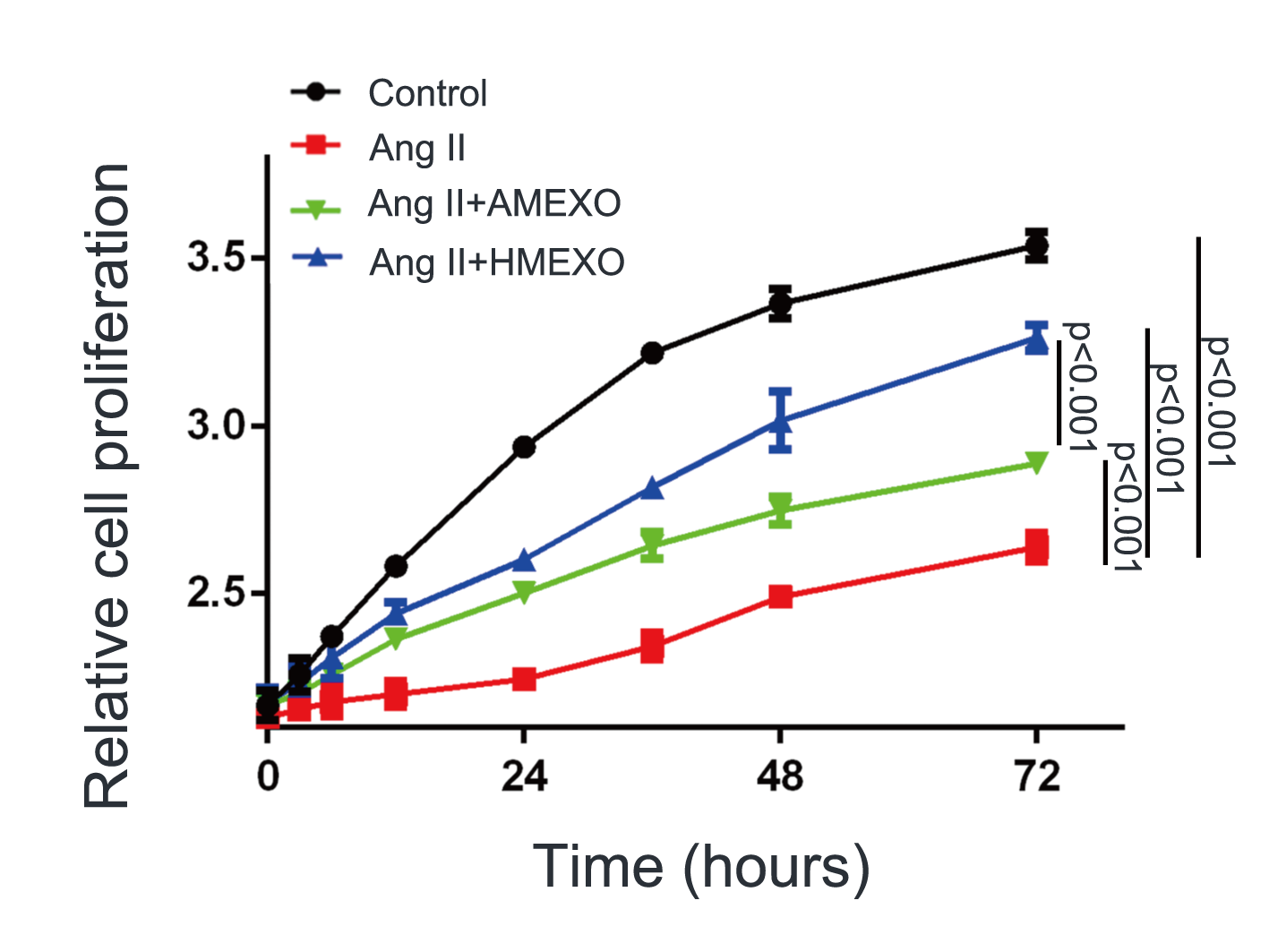


**Supplemental Figure 6. HMEXO more effectively promoted proliferation of VSMCs than AMEXO.** Relative cell proliferation curve of control VSMCs and VSMCs following Ang II, AngII+HMEXO or AngII+AMEXO treatment by cell counting kit 8 assay (n=6 independent experiments). Data are expressed as mean ± SEM. Linear mixed effects model was performed followed by Holm-Sidak multiple comparison test.


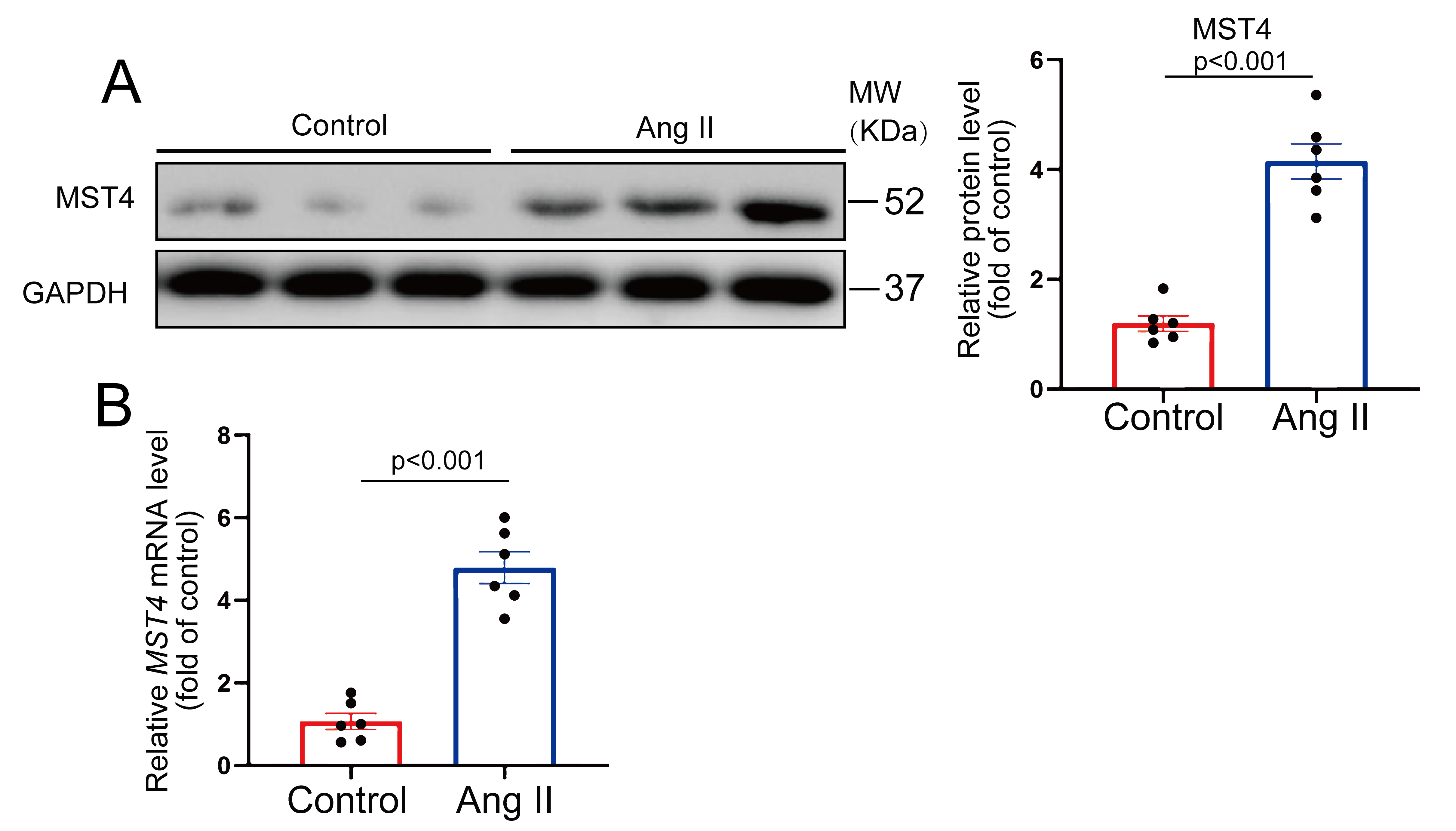


**Supplemental Figure 7. Ang II treatment enhanced protein and mRNA level of MST4 in VSMCs** (A) Western blotting and quantitative analysis of the expression of MST4 in control VSMCs and Ang II-induced senescent VSMCs (n=6 independent experiments). (B) qRT-PCR analysis of *MST4* mRNA expression in control VMSCs and Ang II-induced senescent VSMCs (n=6). Data are expressed as mean ± SEM. Two-tailed Student t test.


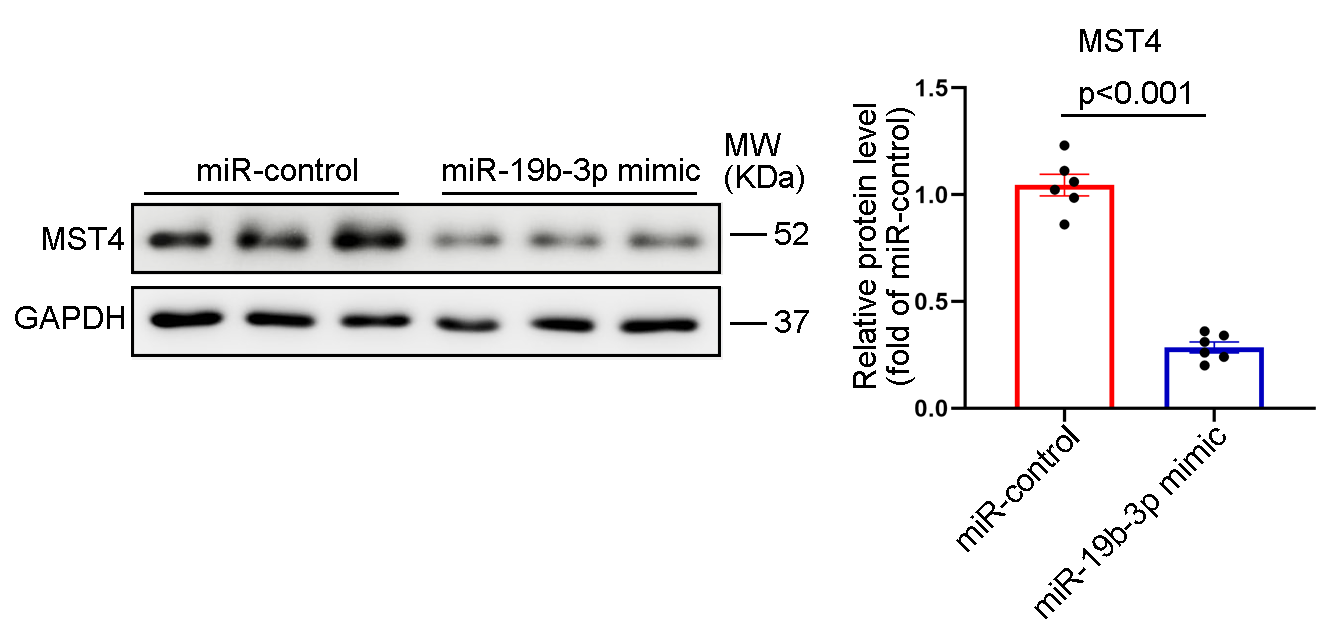


**Supplemental Figure 8. Protein level of MST4 was reduced in mouse VSMCs treated with miR-19b-3p mimic.** Western blotting and quantitative analysis of the expression level of MST4 in control VSMCs and control VSMCs treated with miR-19b-3p mimic (n=6 independent experiments). Data are expressed as mean ± SEM. Two-tailed Student t test.


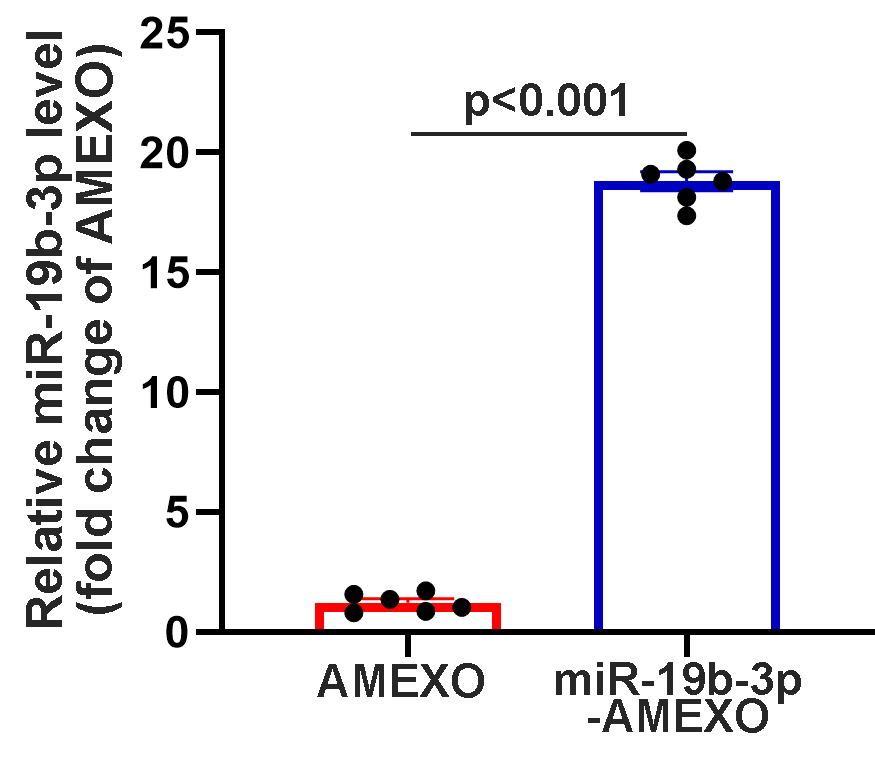


**Supplemental Figure 9. Expression of miR-19b-3p was significantly elevated in miR-19b-3p-AMEXO.** qRT-PCR analysis of miR-19b-3p expression in miR-19b-3p-AMEXO and AMEXO (n=6 independent experiments). Data are expressed as mean ± SEM. Two-tailed Student t test.


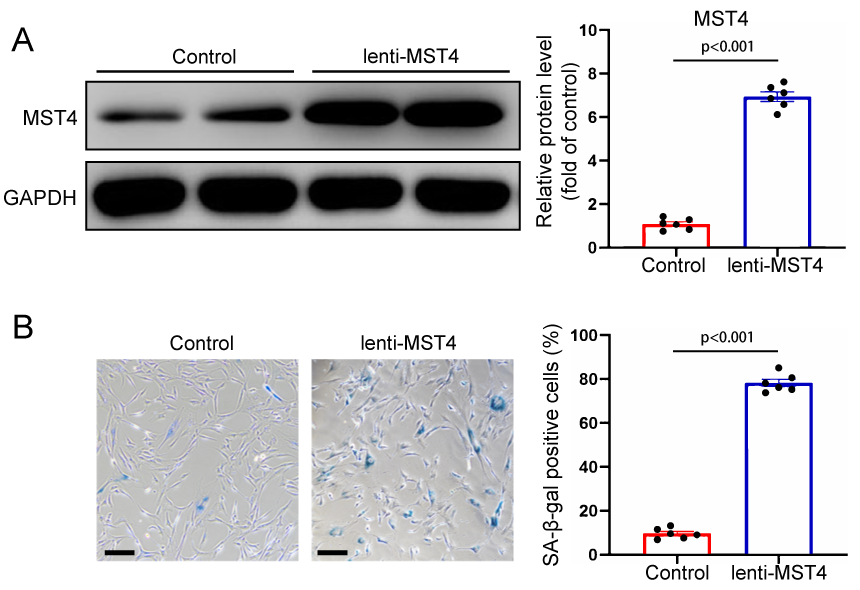


**Supplemental Figure 10. VSMCs transfected with lenti-MST4 elevated senescence.** (A) Western blotting and quantitative analysis of MST4 protein expression in control VSMCs and VSMCs transfected with lenti-MST4 (n=6 independent experiments). (B) Representative images and quantitative analysis of SA-β-gal staining of control VSMCs and VSMCs transfected with lenti-MST4 (n=6 independent experiments). Scale bar: 10 μm. Data are expressed as mean ± SEM. Two-tailed Student t test.


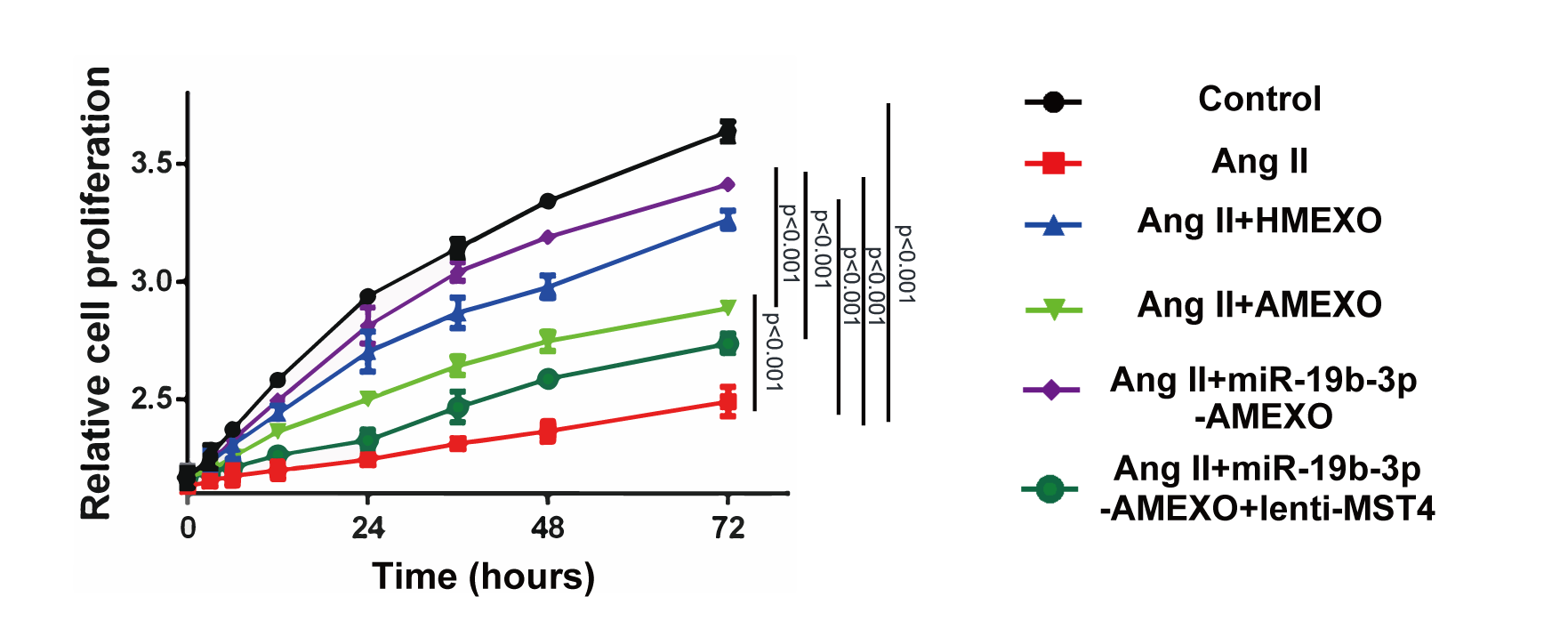


**Supplemental Figure 11. Overexpressed MST4 abrogated the protective effects of miR-19b-3p-AMEXO on senescent VSMCs.** Relative cell proliferation curve of control VSMCs and VSMCs following Ang II, Ang II+HMEXO, Ang II+AMEXO, Ang II+miR-19b-3p-AMEXO or Ang II+miR-19b-3p-AMEXO+lenti-MST4 treatment (n=6 independent experiments). Data are expressed as mean ± SEM. Linear mixed effects model was performed followed by Holm-Sidak multiple comparison test.


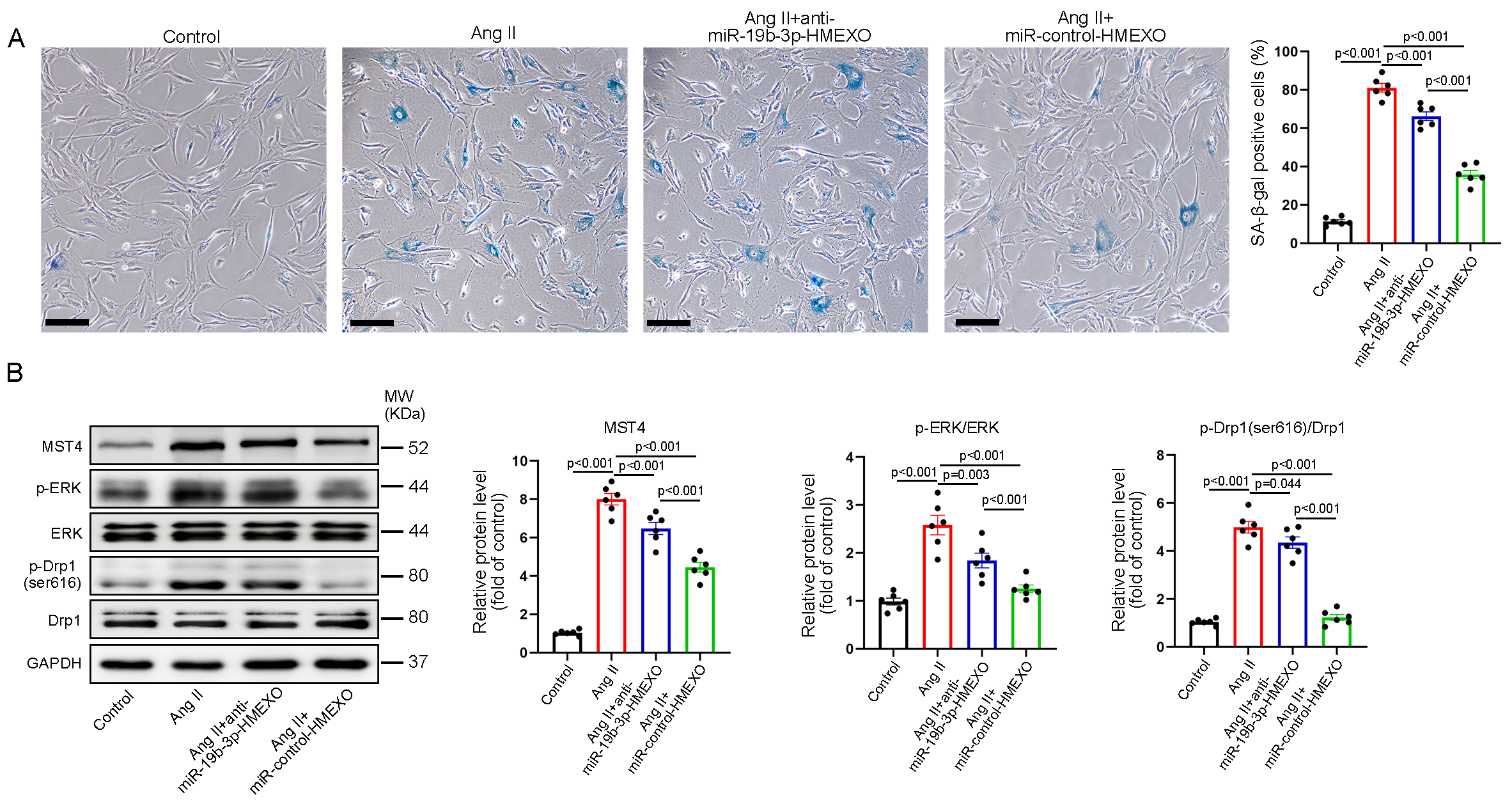


**Supplemental Figure 12. Downregulation of miR-19b-3p expression in HMEXO significantly reduced its ability to inhibit senescence of VMSCs.** (A) Representative images and quantitative analysis of SA-β-gal staining of control VSMCs and VSMCs following Ang II, Ang II+ Ang II+HMEXO+miR-19b-3p inhibitors or Ang II+anti-miR-19b-3p-HMEXO treatment (n=6 independent experiments). Scale bar: 10 μm. (B) Western blotting and quantitative analysis of the expression of MST4, p-ERK, p-Drp1 (Ser616) in control VSMCs and VSMCs following Ang II, Ang II+Ang II+HMEXO+miR-19b-3p inhibitors or Ang II+anti-miR-19b-3p-HMEXO treatment (n=6 independent experiments). Data are expressed as mean ± SEM. One-way ANOVA followed by Holm-Sidak multiple comparison test.


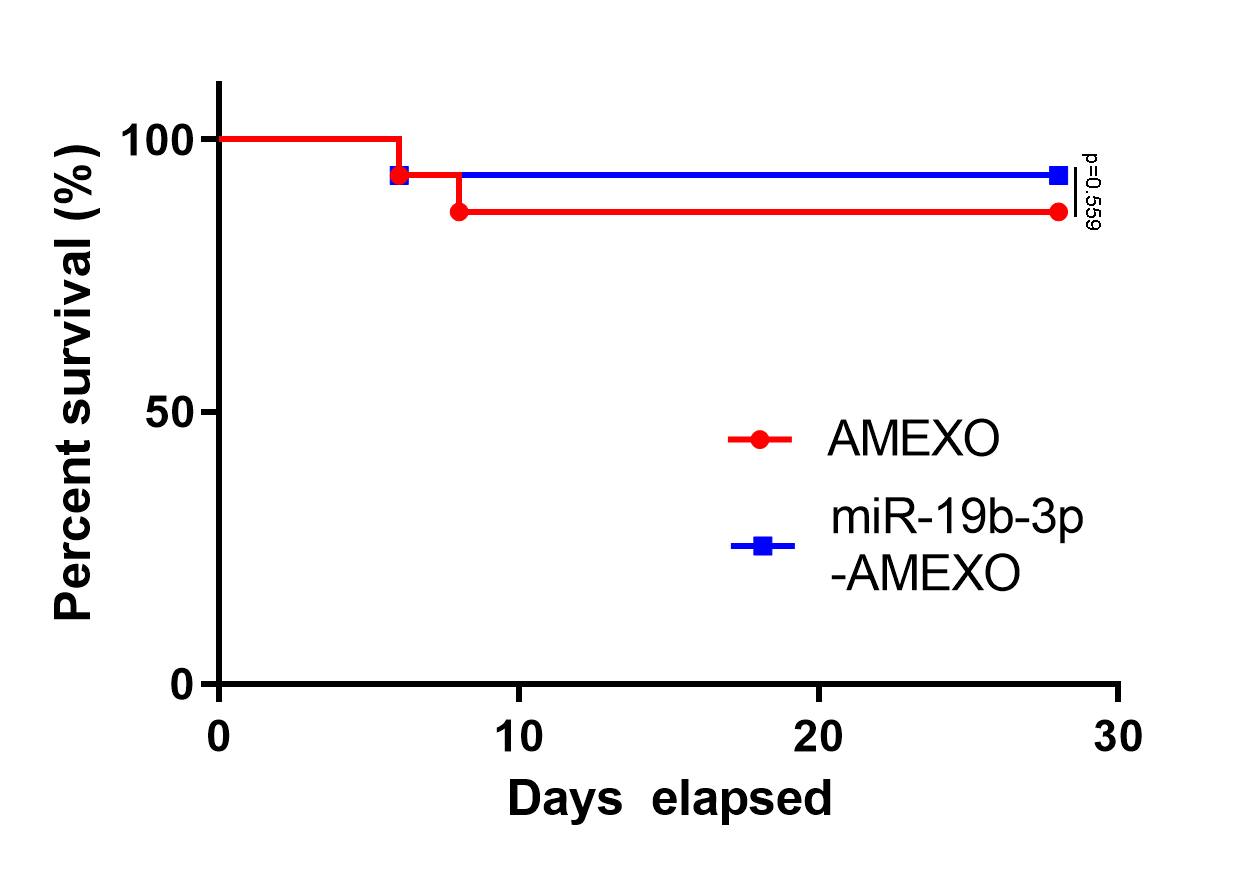


**Supplemental Figure 13. Kaplan-Meier survival curve of** ***ApoE^− /−^* mice and *ApoE^− /−^* mice following AMEXO or miR-19b-3p-AMEXO treatment (n=10 mice). Data are expressed as mean ± SEM. Statistical significance was determined by Log-rank (Mantel-Cox) test.**

**Major Resources Table**

**Animals (in vivo studies)**

| **Species** | **Vendor or Source** | **Background Strain** | **Sex** | **Persistent ID / URL** |
| --- | --- | --- | --- | --- |
| ApoE-/- mice | GemPharmatech Co., Ltd. | C57BL/6J | Male |  |

**Antibodies**

| **Target antigen** | **Vendor or Source** | **Catalog #** | **Working concentration** | **Appl**  **icati**  **on** | **Persistent ID / URL** |
| --- | --- | --- | --- | --- | --- |
| α-SMA | Boster Biological Technology | BM0002 | 2μg/ml | IHC | http://www.boster.com.cn/product/anti-smooth-muscle-actin-a-sma-antibody-monoclonal-1a4_bm0002.html |
| α-SMA | Abcam | ab7817 | 1μg/ml | ICC | https://www.abcam.cn/alpha-smooth-muscle-actin-antibody-1a4-ab7817.html |
| CD68 | Proteintech | 28058-1-AP | 5μg/ml | IHC | https://www.ptgcn.com/products/Cd68-Antibody-28058-1-AP.htm |
| MMP-9 | Proteintech | 10375-2-AP | 5μg/ml | IHC/WB | https://www.ptgcn.com/products/MMP9-Antibody-10375-2-AP.htm |
| Ki67 | Abcam | ab15580 | 1μg/ml | ICC | https://www.abcam.cn/ki67-antibody-ab15580.html |
| CD63 | Abcam | ab134045 | 2μg/ml | WB/IHC | https://www.abcam.cn/cd63-antibody-epr5702-ab134045.html |
| CD81 | Abcam | ab109201 | 2μg/ml | WB | https://www.abcam.cn/cd81-antibody-epr4244-ab109201.html |
| TSG101 | Abcam | ab125011 | 2μg/ml | WB | https://www.abcam.cn/tsg101-antibody-epr7130b-ab125011.html |
| Calnexin | Proteintech | 10427-2-AP | 2μg/ml | WB | https://www.ptgcn.com/products/CANX-Antibody-10427-2-AP.htm |
| p21 | Abcam | ab109199 | 1μg/ml | WB | https://www.abcam.cn/p21-antibody-epr3993-ab109199.html |
| p-Drp1 (ser616) | CST | 3455 | 1μg/ml | WB | https://www.cellsignal.cn/products/primary-antibodies/phospho-drp1-ser616-antibody/3455?site-search-type=Products&N=4294956287&Ntt=3455&fromPage=plp&_requestid=2330438 |
| Drp1 | CST | 14647 | 1μg/ml | WB | https://www.cellsignal.cn/products/primary-antibodies/drp1-4e11b11-mouse-mab/14647?site-search-type=Products&N=4294956287&Ntt=14647&fromPage=plp&_requestid=2330740 |
| p-ERK1/2 | CST | 9101 | 1μg/ml | WB | https://www.cellsignal.cn/products/primary-antibodies/phospho-p44-42-mapk-erk1-2-thr202-tyr204-antibody/9101?site-search-type=Products&N=4294956287&Ntt=9101&fromPage=plp&_requestid=2330797 |
| ERK1/2 | CST | 4695 | 1μg/ml | WB | https://www.cellsignal.cn/products/primary-antibodies/p44-42-mapk-erk1-2-137f5-rabbit-mab/4695?site-search-type=Products&N=4294956287&Ntt=4695&fromPage=plp |
| MST4 | CST | 3822 | 2μg/ml | WB | https://www.cellsignal.cn/products/primary-antibodies/mst4-antibody/3822?site-search-type=Products&N=4294956287&Ntt=3822&fromPage=plp&_requestid=2330944 |
| GAPDH | CST | 2118 | 1μg/ml | WB | https://www.cellsignal.cn/products/primary-antibodies/gapdh-14c10-rabbit-mab/2118?site-search-type=Products&N=4294956287&Ntt=2118&fromPage=plp&_requestid=2330995 |
| Anti-mouse IgG, HRP-linked Antibody | CST | 7076 | 0.0765 µg/ml | WB | https://www.cellsignal.cn/products/secondary-antibodies/anti-mouse-igg-hrp-linked-antibody/7076?site-search-type=Products&N=4294960093+102287+4294956287&Ntt=secondary&fromPage=plp |
| Anti-rabbit IgG, HRP-linked Antibody | CST | 7074 | 0.0765 µg/ml | WB | https://www.cellsignal.cn/products/secondary-antibodies/anti-rabbit-igg-hrp-linked-antibody/7074?site-search-type=Products&N=4294960093+102287+4294956287&Ntt=secondary&fromPage=plp |
| Donkey anti-Rabbit IgG (H+L) Highly Cross-Adsorbed Secondary Antibody, Alexa Fluor 555 | ThermoFisher | A-31572 | 2μg/ml | IHC/ICC | https://www.thermofisher.cn/cn/zh/antibody/product/Donkey-anti-Rabbit-IgG-H-L-Highly-Cross-Adsorbed-Secondary-Antibody-Polyclonal/A-31572 |
| Donkey anti-Mouse IgG (H+L) Highly Cross-Adsorbed Secondary Antibody, Alexa Fluor 488 | ThermoFisher | A-21202 | 2μg/ml | IHC/ICC | https://www.thermofisher.cn/cn/zh/antibody/product/Donkey-anti-Mouse-IgG-H-L-Highly-Cross-Adsorbed-Secondary-Antibody-Polyclonal/A-21202 |
| Mouse (G3A1) mAb IgG1 Isotype Control | CST | 5415 | 2μg/ml | IHC/ICC | https://www.cellsignal.cn/products/primary-antibodies/mouse-g3a1-mab-igg1-isotype-control/5415?site-search-type=Products&N=4294960093+4294960090+102236+4294956287&Ntt=igg&fromPage=plp |

**DNA/cDNA Clones**

| **Clone Name** | **Sequence** | **Source / Repository** | **Persistent ID / URL** |
| --- | --- | --- | --- |
| MST4 | atggcccactcgccggtggctgtccaagtgcctgg  gatgcagaataacatagctgatccagaagaactg  ttcacaaaattagagcgcattgggaaaggctcatt  tggggaagttttcaaaggaattgataaccgtaccc  agcaagtcgttgctattaaaatcatagaccttgagg  aagccgaagatgaaatagaagacattcagcaaga  aataactgtcttgagtcaatgtgacagctcatatgta  acaaaatactatgggtcatatttaaaggggtctaaa  ttatggataataatggaatacctgggcggtggttcag  cactggatcttcttcgagctggtccatttgatgagttc  cagattgctaccatgctaaaggaaattttaaaaggtc  tggactatctgcattcagaaaagaaaattcaccgag  acataaaagctgccaatgtcttgctctcagaacaagg  agatgttaaacttgctgattttggagttgctggtcagc  tgacagatacacagattaaaagaaatacctttgtggg  aactccattttggatggctcctgaagttattcaacagtc  agcttatgactcaaaagctgacatttggtcattgggaa  ttactgctattgaactagccaagggagagccacctaa  ctccgatatgcatccaatgagagttctgtttcttattcc  caaaaacaatcctccaactcttgttggagactttactaa  gtcttttaaggagtttattgatgcttgcctgaacaaagat  ccatcatttcgtcctacagcaaaagaacttctgaaacac  aaattcattgtaaaaaattcaaagaagacttcttatctgact  gaactgatagatcgttttaagagatggaaggcagaagg  acacagtgatgatgaatctgattccgagggctctgattcg  gaatctaccagcagggaaaacaatactcatcctgaatgg  agctttaccaccgtacgaaagaagcctgatccaaagaa  agtacagaatggggcagagcaagatcttgtgcaaaccct  gagttgtttgtctatgataatcacacctgcatttgctgaact  taaacagcaggacgagaataacgctagcaggaatcaggc  gattgaagaactcgagaaaagtattgctgtggctgaagcc  gcctgtcccggcatcacagataaaatggtgaagaaactaa  ttgaaaaatttcaaaagtgttcagcagacgaatccccc |  |  |

**Cultured Cells**

| **Name** | **Vendor or Source** | **Sex (F, M, or unknown)** | **Persistent ID / URL** |
| --- | --- | --- | --- |
| VSMC | Guangdong Provincial People’s Hospital | M |  |
| ADMSC | Guangdong Provincial People’s Hospital | M |  |
| HEK 293T/17 | ATCC | unknown | https://www.atcc.org/products/crlͲ  11268 |

**qPCR primer information**

| **Gene** | **Forward** | **Reverse** |
| --- | --- | --- |
| *MST4* | TTCGAGCTGGTCCATTTGATG | TGAATGCAGATAGTCCAGACCT |
| has-miR-19b-3p | CGAGCCGGATCCGTTAG | CGACTAGGCTTCGCTAGA |
| hsa-miR-133a-3p | ACACTCCAGCTGGGTTTGTCCCCTTCAAC | TGGTGTCGTGGAGTCG |
| hsa-miR-30c-5p | GCGCGTGTAAACATCCTACACT | AGTGCAGGGTCCGAGGTATT |
| *Il6* | ACAAAGAAATGATGGATGCTACC | GTATCTCTCTGAAGGACTCTGG |
| *Ccl2* | TCTCTCTTCCTCCACCAC | CTCTCCAGCCTACTCATTG |
| *Mmp-2* | TGGAATGCCATCCCTGATAA | AGCCCAGCCAGTCTGATTTG |
